# Team reasoning—Experimental evidence on cooperation from centipede games

**DOI:** 10.1371/journal.pone.0206666

**Published:** 2018-11-28

**Authors:** Johann Graf Lambsdorff, Marcus Giamattei, Katharina Werner, Manuel Schubert

**Affiliations:** Faculty of Business Administration and Economics, University of Passau, Passau, Germany; Middlesex University, UNITED KINGDOM

## Abstract

Previous laboratory studies on the centipede game have found that subjects exhibit surprisingly high levels of cooperation. Across disciplines, it has recently been highlighted that these high levels of cooperation might be explained by “team reasoning”, the willingness to think as a team rather than as an individual. We run an experiment with a standard centipede game as a baseline. In two treatments, we seek to induce team reasoning by making a joint goal salient. First, we implement a probabilistic variant of the centipede game that makes it easy to identify a joint goal. Second, we frame the game as a situation where a team of two soccer players attempts to score a goal. This frame increases the salience even more. Compared to the baseline, our treatments induce higher levels of cooperation. In a second experiment, we obtain similar evidence in a more natural environment–a beer garden during the 2014 FIFA Soccer World Cup. Our study contributes to understanding how a salient goal can support cooperation.

## Introduction

Cooperation has fascinated scientists from many disciplines for a long time. For the sake of collective welfare, humans are frequently willing to incur personal costs even if they could unilaterally benefit from defection. This has often been related to altruism towards genetically related group members or to long-term gains that can be reaped in repeated interaction [1]. But laboratory experiments have shown that levels of cooperation are high even when people play anonymously with partners that are unknown and unrelated to them and even when interaction is one-shot such that cooperation promises no future benefits from repeated interaction [2, 3, 4].

There are many real-world examples of situations in which humans can advance together if they invest in turns, but at the same time face the risk that the other person might defect. These examples range from business situations, where the implementation of a business plan involves various milestones that must be met by specialized partners in an alternating fashion, to academia, where temptations to steal ideas and data must be overcome in light of sequential investments of human resources. They include personal situations, such as friendships or romantic relationships in which people alternately invest by supporting each other, and day-to-day exchange like tipping after having been served well in a restaurant. In these and similar situations, cooperation is more efficient for the group or society as a whole, but one person could reap the benefits by acting selfishly.

The centipede game nicely reflects such a trade-off between self-interest and mutual benefit in sequential interaction. As a multistage trust game with alternating offers, it mirrors the dynamics and the reciprocal nature of cooperative interaction. A two-player version with exponentially increasing payoffs and four stages is described in the following: in the first stage, player A can take €8 and leave €2 to player B or pass the decision to B, in which case payoffs double and B faces a similar decision. If B takes in the second stage, she obtains €16 and A gets €4. If she passes, she lets A decide and payoffs are doubled again. The game continues for 4 stages, promising up to €128 to A and €32 to B. The longer players pass, the higher their joint payoffs become. This reflects that cooperation is efficient on the long run. But in each stage, the player who passes faces the risk of losing half of her payoff. Taking guarantees a safe payoff while passing increases the joint welfare.

The centipede game has been studied in the laboratory with a variety of design choices. For example, joint payoffs stay constant in some variants and increase linearly or exponentially across stages in others. Different numbers of stages have been investigated, ranging from a minimum of three to a maximum of 20. While the standard version involves two players, variants have been run with up to five. Some researchers have run the game repeatedly so as to allow for learning, while others have recruited participants that are known to excel in strategic thinking, for a recent review see [5].

The centipede game is particularly interesting, because most empirical findings strongly contradict game theoretical predictions. The unique subgame-perfect Nash equilibrium–the situation in which no player has a unilateral incentive to change her strategy–predicts that no player should ever pass [6, 7]. This logic follows from backward induction, i.e. from reasoning through the game from its end. Passing reduces B’s payoff in the final stage, inducing an individually optimal choice in favor of taking. Knowing this, A will prefer to take in the penultimate stage and so forth. However, with only few exceptions [8, 9, 10], the experimental evidence from the laboratory reveals a remarkable consistency in regard to two findings. First, a vast majority of players passes in the first stage. Only a very minor share of the players, in some cases none, takes at the outset. Second, the level of passing decreases across stages in most versions of the centipede game. Non-zero levels of passing robustly persist across the first stages, and some games end without taking [11–15]. These robust findings are puzzling not only because they contradict the theory but also because the reasons for this departure from the subgame-perfect Nash equilibrium remain unresolved.

Some studies refer to other-regarding preferences such as altruism and reciprocity [16–20] to explain the findings. Passing should be preferred by players who derive utility from the payoff obtained by others, potentially contingent on the expectation that these will also act reciprocally. But a recent study on the centipede game does not find support for reciprocal motives to explain the data [21]. This points to another idea, namely a motive to maximize joint payoffs [19, 21], and to recent theories on team reasoning: Scientists from economics [22–31], philosophy [32–35], evolutionary anthropology [36], and psychology [37–38] have recently contributed to an understanding of this driver of cooperation, sometimes also referred to as we-thinking or joint intentionality. These studies share the thought that cooperation is not fully explained by theories of individual rational maximization.

Certain circumstances can induce subjects to think as a team rather than as an individual. Thinking as a team refers to the question “what should we do?” rather than “what should I do?”. Subjects then infer the joint action that all should take in order to achieve a mutual advantage. They develop a shared plan, expect others to make choices in line with this plan and are willing to make the choices that are necessary for achieving the joint goal. Team reasoning is likely to occur under circumstances where a joint goal is made salient. Bacharach ([22]: p. 144) argues that team reasoning is supported if “constitutive features of the basic game make collective identity salient“. This may be achieved if attention is directed towards the joint goal. Tomasello ([36]: p. 44) investigates cooperation from the perspective of an evolutionary anthropologist and argues with reference to primates:

“Underlying this coordination is … some notion of common ground, in which each individual … can attend to his partner’s attention, his partner’s attention to his attention, and so forth… This is … the ‘top-down’ version of joint attention because it is directed by joint goals.”

The level of team reasoning might depend on the payoffs that each end node of the game tree allocates to players. Some games promise higher rewards for cooperation, while others are more risky for cooperators or more tempting for uncooperative subjects. Attempts have thus been made to infer the level of team reasoning from payoffs [5, 21, 26, 28, 30–31]. What has, to the best of our knowledge, not been investigated yet is whether variations in the experimental design can make a difference even if they leave expected payoffs unaffected. For this purpose, we ran a standard centipede game (baseline) and two further treatments where we increase the salience of a joint goal. In the first treatment, subjects play a probabilistic variant of the centipede game where both players are made aware of a joint goal (a joint payoff of €200 in our experiment), with the probability of reaching the goal increasing across stages. To increase the salience further, we describe the centipede game as a game of soccer in the second treatment. We argue that our manipulations make it more likely that subjects think as a team rather than as an individual. Indeed, we find cooperation to increase in these treatments.

We further tested the soccer treatment in a natural environment. We ran this experiment at two public viewing events in Bavarian beer gardens with a live-broadcast of a soccer game on a large screen. With soccer fans as our sample, we observe that the high level of cooperation persists. Altogether, we find that our design manipulations are capable of increasing cooperation and that theories of team reasoning are likely explanations for our findings.

## Experiment 1

**Fig 1:**
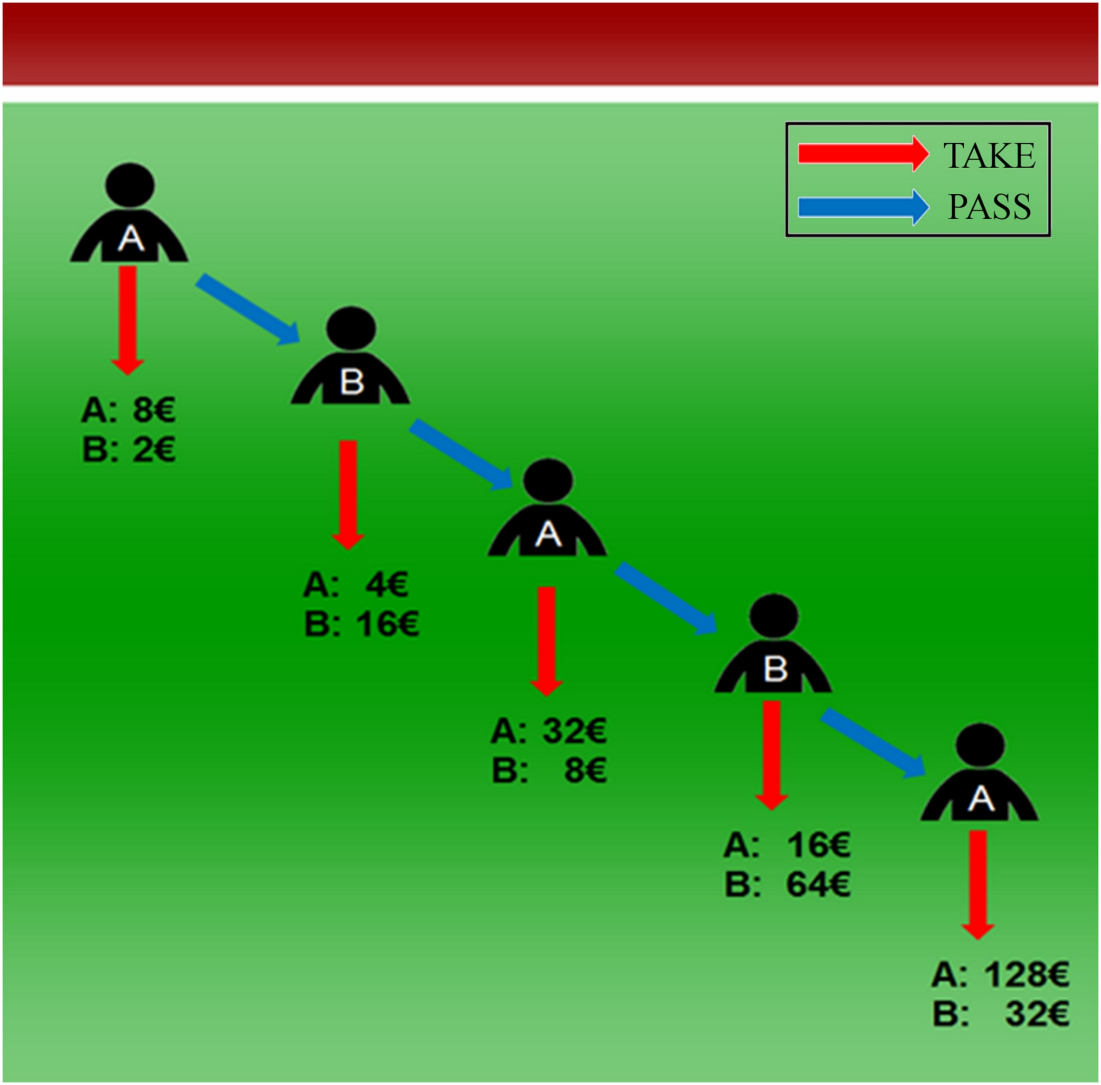
Illustration of the centipede game. Passing doubles joint payoffs and hands over the larger share to the other player.

In the baseline, subjects played a standard (exponential) four-stage centipede game as described in the introduction and as depicted in Fig 1. The game was played only once. Players in turn decided whether to take or pass, with taking being illustrated by red arrows and passing by blue arrows. Taking ended the game and secured a player the higher payoff, while joint payoffs doubled with each decision to pass. At the last stage, player A had no decision to make but could only take. Nonetheless, Fig 1 displays player A in order to minimize differences to the soccer treatment that will be explained below. In two treatments, we manipulated the presentation of the payoffs and the framing of the game to gradually increase the salience of the joint goal.

**Fig 2:**
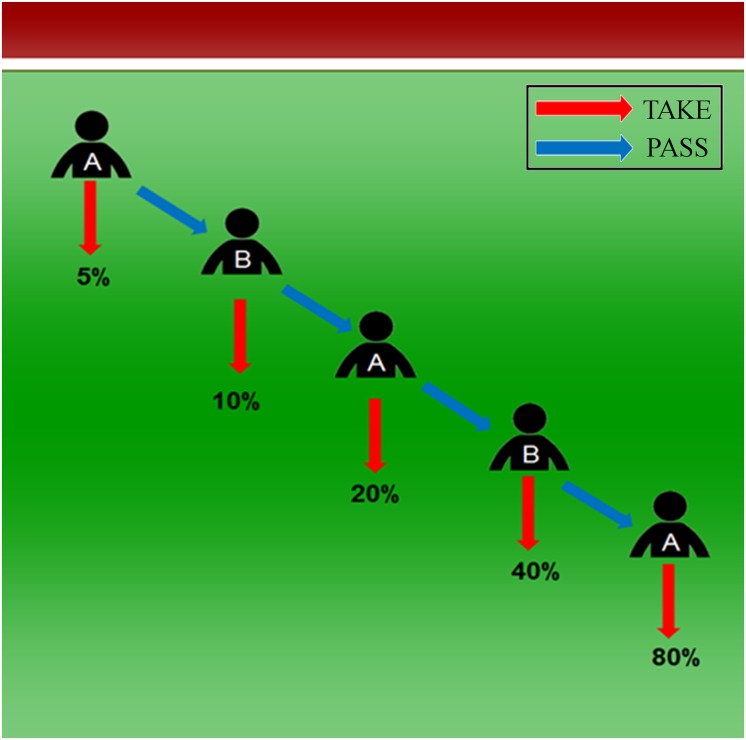
Illustration of the probabilistic variant of the centipede game. Passing doubles the group’s chance of achieving a success while taking secures an individual the possibility to receive the higher payoff, which is €160 for the subject who takes compared to only €40 for the other player.

In the treatment “Probabilistic”, we introduced probabilistic instead of deterministic payoffs, as shown in Fig 2. Instead of increasing the joint payoff, passing in the treatment “Probabilistic” increased the group’s chance of achieving a success. In case of a success, the subject who took would receive €160 and the other subject would receive €40. The chance to achieve a success started at 5% in the first stage and doubled with each pass up to 80% where the game would end, so that expected payoffs were equivalent to the payoffs in the deterministic baseline “Centipede”. Our variation shows some similarity to the one employed by [39], where payoffs are expressed as (alternating) percentages of the joint payoff that would be achieved in the last stage. They do not find this variation to impact behavior. In our approach, however, the percentages do not express shares of a joint payoff, but the probability of a joint success that is identical for both players.

For risk neutral players, it should make no difference whether the non-probabilistic “Centipede” or the probabilistic variant “Probabilistic” is played. What should make a difference is that the increasing probabilities in “Probabilistic” point to a joint goal and an associated shared plan: repeated passing in order to increase the probability of a success. Participants in “Centipede”, as in any regular centipede game, face the task of inferring the joint goal from the payoff structure, which does not make this joint goal and the related shared plan salient. If the salience of a joint goal increases cooperation, we thus expect the percentage of players who pass in “Probabilistic” to exceed that in “Centipede”.

**Fig 3:**
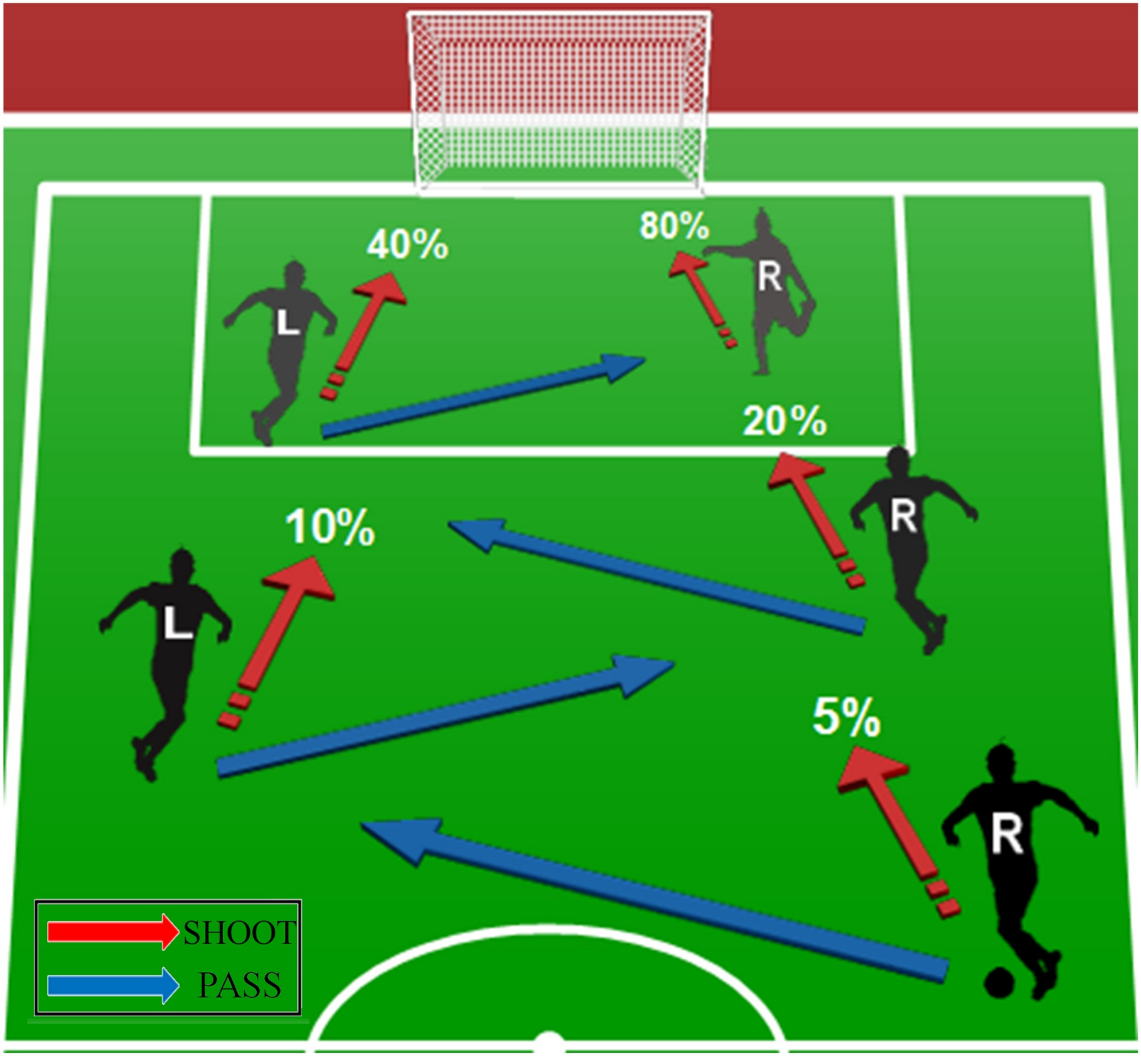
Illustration of the centipede game framed as a game of soccer. Passing doubles the team’s chance of scoring a goal while shooting secures an individual the possibility to receive the higher payoff, which is €160 for the scorer compared to only €40 for the other player.

In the treatment “Soccer”, we described the game in an intuitive form as a situation in a soccer game, as shown in Fig 3. The roles, this time labelled L and R, were described as left wing (L) and right wing (R). Both roles were informed that R has the ball in the beginning. In the first stage, R was asked to decide whether to take a shot at the goal or to pass the ball to L. In case of shooting, R would score a goal with a 5% probability and the game would end.

Like in the treatment “Probabilistic”, the probability of scoring a goal doubled with every pass, up to 80% where R would shoot for sure and the game would end. This doubling corresponds to shot conversion rates in real soccer games. For example, [40] show that conversion rates are around 2–3 percent for a distance of 30–40 yards, increase to about 7 percent for 20–30 yards, 13 percent for 10–20 yards and reach 30 percent for distances below 10 yards. Like in “Probabilistic”, the successful scorer would earn €160 while the other player would receive only €40.

This description as a game of soccer supports the salience of a joint goal even more. All look at the same object (the goal), imagine an obstacle (the rival team), notice an instrument for scoring (the ball), are heading in the same direction on the soccer pitch and observe the cooperative actions and a shared plan (passing the ball and running towards the goal). Table 1 summarizes the baseline and the treatments.

**Table 1:** Overview of the baseline and the two treatments.

	Description	Condition	Observations
Centipede	Regular centipede game	Non-probabilistic	111
Probabilistic	Game with probabilities	Probabilistic	121
Soccer	Game of soccer	Probabilistic	91

Our sample was a classroom with a large audience of university students who had previously attended a lecture in microeconomics (with no reference to game theory in the lecture so far). 323 students completed the game. The sample comprised students from various disciplines such as business administration and economics (64%), political science (17%), cultural studies (7%) and teaching (8%). Subjects participated only either in the baseline or in one the treatments (between-subject design). 56% were female and around 49% were below 20 years, 48% between age 21 and 24 and 3% older than 24. Participants were informed at the beginning of the lecture that they had the possibility to participate in a classroom experiment with their smartphones. Flyers with detailed instructions had been circulated on the tables before the lecture. The experiment was conducted at the end of the regular lecture and students were informed that the experiment was not part of the lecture. This gave them the possibility to leave the lecture hall before the experiment. Flyers informed participants that the data were being collected for research purposes and about standard anonymity and blindness procedures. The full set of instructions can be found in the supporting information. Consent to participation was given uno actu by opening a website via a QR code or an URL. Participation was completely anonymous and no (identifiable) personal data were collected.

A public announcement was made, explaining that each participant would be randomly matched to another participant from the audience, that their payoffs depended on their own decisions and those of this other participant, that ten pairs of participants would be randomly chosen to receive actual payoffs at the end of the experiment, and that these payoffs would be paid out in private by a third person who did not know the game. They were also advised to read the written instructions carefully and to follow the step-by-step instructions on the smartphones. Participants were publicly instructed to open a website with their smartphones where they would be matched in pairs of two, where roles (A or B; R or L) would be assigned and where they would be guided through the game step-by-step.

The experiment was programmed with classEx [41]. This program provides smartphone users with immediate access to the game without having to register. Smartphone coverage in Germany is currently close to 80%, suggesting that participation was possible for most students. The software also allows for random and blind matching and assignment of roles in the experiment. To ensure a maximum understanding of the instructions, all participants had to answer comprehension questions correctly before being able to proceed. Ten pairs of participants were randomly chosen to obtain the designated payoffs. In total, €1020 were paid out by a third person, who did not know the game. These payoffs were made in private outside the classroom at two separate locations for A (R) and B (L) in order to avoid collusion and to ensure conditions of double-blindness. The game lasted about 5 minutes. On average, 5 additional minutes were required for reading the instructions.

We used the strategy method, i.e. elicited responses to (almost) all decision nodes to keep data traffic minimal. As reported by [15], the strategy method might bring about behavior that differs from the game method in games where subjects make many contingent choices, which is the case in a centipede game. For example, B in stage 4 will recognize that her choice is contingent on A passing in stages 1 and 3 and her own choice to pass in stage 2. As reviewed by [5], studies using the strategy method for the centipede game tend to report higher levels of cooperation. Our approach, however, simplifies the strategy method and avoids the complexity of contingent choices. Unlike in [13], our players do not decide at the outset at which decision node to take. Rather, like in [42], participants were guided through the experiment step-by-step, as if they were interacting with another participant. They decided sequentially for each stage whether to take, contingent on passing in previous stages. If they decided to take in a certain stage, they did not have to make any further decision, as if they were playing game method. Matching of participants into pairs took place at the end of the experiment for the winning participants. This design choice eliminates problems with participants dropping out during the course of the experiment, a problem that we had experienced in other previous experiments with users of smartphones.

We also elicited beliefs and asked participants in each stage whether they expected the other participant to take or pass in the subsequent stage. After the experiment, subjects had to complete a questionnaire where we asked for standard demographics (gender, age and major), the self-assessed level of risk seeking, whether they had seen their neighbor’s instructions, communicated with other participants and if they had previously participated in any experiments of this study. We also asked for their general mood and their agreement with the statement: “An individual should subordinate himself/herself to the good of the community”. The exact wording of the questions can be found in the supporting information. The baseline and the two treatments were run simultaneously in the lecture hall. Subjects in the baseline and the different treatments were seated in different blocks of the lecture hall to avoid design contamination. Only 2 subjects noted that they had different instructions than others. 16 subjects noticed similar instructions and 305 did not take notice of others’ instructions.

## Results and econometric approach

**Fig 4:**
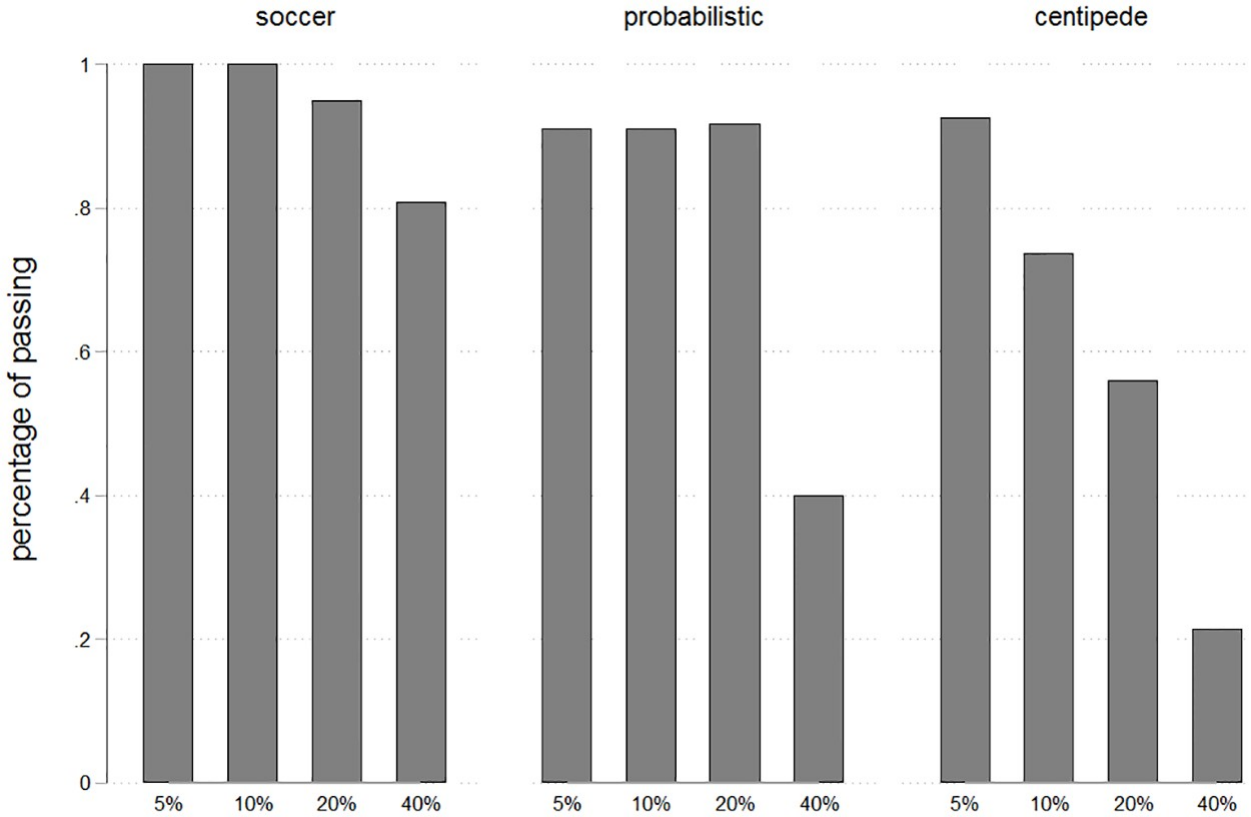
Percentages of passing by treatment. Percentages denote the share of participants who decide to pass among all those who have reached a given stage. As can be seen, percentages are highest in “Soccer”, intermediate in “Probabilistic” and lowest in “Centipede”.

The number of passes could lie between 0 and 2 for each player. The average number of passes was highest in “Soccer” (1.81 for L and 1.95 for R), intermediate in “Probabilistic” (1.27 for B and 1.74 for A) and lowest in “Centipede” (0.89 for B and 1.44 for A). Fig 4 shows the resulting (conditional) percentages of players who passed in each stage. Percentages are highest in “Soccer” where all subjects pass in stages 1 and 2. They are at an intermediate level in “Probabilistic” and least frequent in “Centipede”. This provides supportive evidence for the idea that salience of a joint goal advances team reasoning and cooperation.

Our percentages of players who passed in “Centipede” are quite standard for short centipede games [5] and similar to those in the seminal paper by McKelvey and Palfrey [14]. We used their data from a 4-stage centipede game and calculated conditional percentages of passing for the first repetition (page 834 and 835, column 3 in each table). The conditional percentages of passing are 100%, 75%, 38% and 27%. We are thus able to replicate standard findings for centipede games.

To determine the significance of our graphical findings, we run ordered logit regressions with the total of our 323 observations from the classroom. The dependent variable in Table 2, regressions 1, 2 and 4, is the number of passes by each participant, which is either 0, 1 or 2. We use dummy variables for the treatments “Soccer” and “Probabilistic”, while “Centipede” serves as the baseline in all our regressions in Table 2.

**Table 2:** Ordered logit regressions on subject’s number of passes.

	regression 1	regression 2	regression 3	regression 4
Dependent variable	Number of passes (ranging from 0 to 2)		Expected number of passes	Number of passes
**Treatment variables**				
Probabilistic	1.21*** (4.4)	1.29*** (4.6)	0.74** (2.8)	1.07*** (3.5)
Soccer	3.00*** (7.7)	3.00*** (7.6)	1.76*** (5.4)	2.49*** (6.0)
**Game variables**				
Player B or left wing	-1.74*** (-6.4)	-1.71*** (-6.2)	1.00*** (4.2)	-2.62*** (-7.7)
Expected number of passes				2.09*** (7.3)
**Individual characteristics**				
Risk-seeking		0.08 (0.6)		-0.06 (-0.5)
Communicated		0.17 (0.3)		0.12 (0.2)
“Subordinate to the good of the community”		-0.11 (-0.8)		-0.01 (-0.09)
Observations	323	315	323	315
(pseudo) *R*^2^	0.19	0.20	0.09	0.32

*t*-statistics in parentheses,

** *p* < 0.01,

*** *p* < 0.001

We observe positive coefficients for the treatment “Probabilistic” across all regressions in Table 2, which indicates that participants pass more often in this treatment than in “Centipede”. The coefficients for “Soccer” are even higher than those for “Probabilistic”. In regression 1, for example, the coefficient of 3.00 is significantly higher than the 1.21 for “Probabilistic” (Wald Test, X^2^ = 21.91 p = 0.0000, two-tailed). The reference to a joint success in “Probabilistic” thus increases passing in comparison to the confrontation with expected payoffs in “Centipede” and the soccer frame increases passing even further.

Coefficients in logit regressions are not easy to interpret, so we will attempt an intuitive illustration. Only 1/3 of the participants pass twice in “Centipede” while 2/3 pass less often. The odds ratio of these two values is (1/3)/(2/3) = 1/2. In “Probabilistic”, about 3/5 of the participants make two passes while 2/5 pass less often. The odds ratio of these values is 3/2. The change from 1/2 to 3/2 amounts to a multiplication by 3. Thus, being in the probabilistic treatment increases the odds ratio by 3. A similar calculation can then be made for passing at least once rather than making 0 passes. Both these calculations yield values around 3. While controlling for further explanatory variables, Table 2 estimates this value to be 3.35 and reports its natural logarithm, which amounts to 1.21.

Further data on game variables are included in Table 2. We control for the position with the variable “Player B or left wing”. Coefficients of -1.74 and -1.71 (regressions 1 and 2) indicate lower passing rates for B (or L), presumably related to B’s lower chances of getting the decision (or the ball) back due to her position in the game tree.

In regression 2 and 4, we control for the additional self-reported data on subjects’ individual characteristics described above. Risk-seeking might induce a difference between the probabilistic treatments and the non-probabilistic “Centipede”. But we do not find support for such an impact and inclusion of this variable makes no difference to our findings. We asked whether participants had communicated with other smartphone users during the experiment. Again, this variable was immaterial to our results. We also included the agreement to the statement “An individual should subordinate himself/herself to the good of the community” into the regression with low values indicating a strong agreement. This self-assessed attitude has no predictive power and does not impact our findings. In addition, we ran regressions that included self-assessed data on whether subjects had participated in the experiment before, and further demographic data on gender, age group and field of study. We do not report details, owing to the fact that these variables were insignificant and did not affect our findings.

We also elicited beliefs regarding the other player’s decisions. Table 2 shows the regression results with the expected number of passes as the dependent variable in regression 3. The variable “Left wing” has a positive impact on the expected number of passes with a coefficient of 1.00. This is because left wings’ beliefs refer to earlier stages of the game (stage 1 and 3) than right wings’ beliefs (stages 2 and 4) and passing is thus more likely. As shown in regression 3, the expected number of passes is highest in our treatment “Soccer”, with a coefficient of 1.76, and also positive but lower in “Probabilistic”. This shows that both treatments had an impact on expectations similar to that on actual behavior. A salient joint goal increases expectations of cooperative behavior.

This raises the question whether treatments with a salient joint goal impact behavior largely via changing beliefs. The high cooperation in our treatments may result because the salient goal increases expectations of cooperation. These expectations may then induce some participants to best respond by passing in early stages. Indeed, as shown in regression 4, Table 2, the expected number of passes obtains a positive coefficient of 2.09 and the (pseudo) R^2^ of 0.32 reveals that the explanatory power of the regression increases substantially. Controlling for this impact in regression 4, we observe that coefficients for treatment variables are slightly lower compared to those in regression 2. Yet, the coefficients for the treatments remain strong and highly significant. Thus, a salient joint goal does not only advance cooperation indirectly by changing beliefs. It has a direct impact on the decision to pass.

## Experiment 2

To confirm the validity of our results, we tested the novel soccer frame in a second experiment in a more natural environment and with a more representative sample. As explained in [43], this experiment was run in July 2014 at public viewing events in two different Bavarian beer gardens during the quarterfinal (Germany vs. France) and the final (Germany vs. Argentina) of the Soccer World Cup. Altogether, 134 subjects participated. 49% of the sample was female. 9% of participants were under the age of 20, 84% between 20 and 30 years old and around 7% older than 30.

This environment is “natural” for at least three reasons. First, participants covered all age groups and included working people (24%) and pupils (4%). Students (72%) were nevertheless overrepresented relative to the German population average. Second, participants did not enroll for participation in a microeconomic lecture as in experiment 1 but attended due to their interest in soccer and also potentially participated because the instructions referred to a game of soccer. Third, participants were not located in an artificial laboratory or attending an academic lecture. Therefore, the environment can additionally serve as a robustness check for our results with respect to selection issues.

Visitors at the beer gardens were informed about the experiment at the entrance and received a flyer with detailed instructions as in the classroom. They were made aware of the chance to participate through public announcements with a loudspeaker. The game was fully explained via loudspeaker, too. The game started after the national anthems had been played, just before the kick-off, and subjects had the entire first half of the soccer game to start the experiment and make their decisions. We ensured that subjects paid attention by asking two comprehension questions which had to be answered correctly in order to participate.

Participation was voluntary. Flyers informed participants that data were being collected for research purposes and about standard anonymity and blindness procedures. Consent to participation was given uno actu by opening a website via a QR code or an URL. Subjects could stop the experiment at any time. Participation was completely anonymous and no (identifiable) personal data were collected. As the experiment was run in a beer garden, we also elicited self-reported drunkenness. 92% of participants reported to be sober or almost sober. Again, we asked for mood, risk-aversion, communication with other participants and previous participation in experiments that were part of the study. The full set of instructions can be found in the supporting information.

Eight pairs were randomly chosen of which three had scored a goal, such that €600 were paid out. Payoffs in the field were made publicly. Observers in the field could neither infer choices nor roles from payoffs. For example, a payoff of €160 could be obtained by R players who had received a pass in the 4th stage and then shot automatically after having been cooperative to the full extent. It could equally be achieved by a player who shot immediately. This preserves conditions of anonymity.

**Fig 5:**
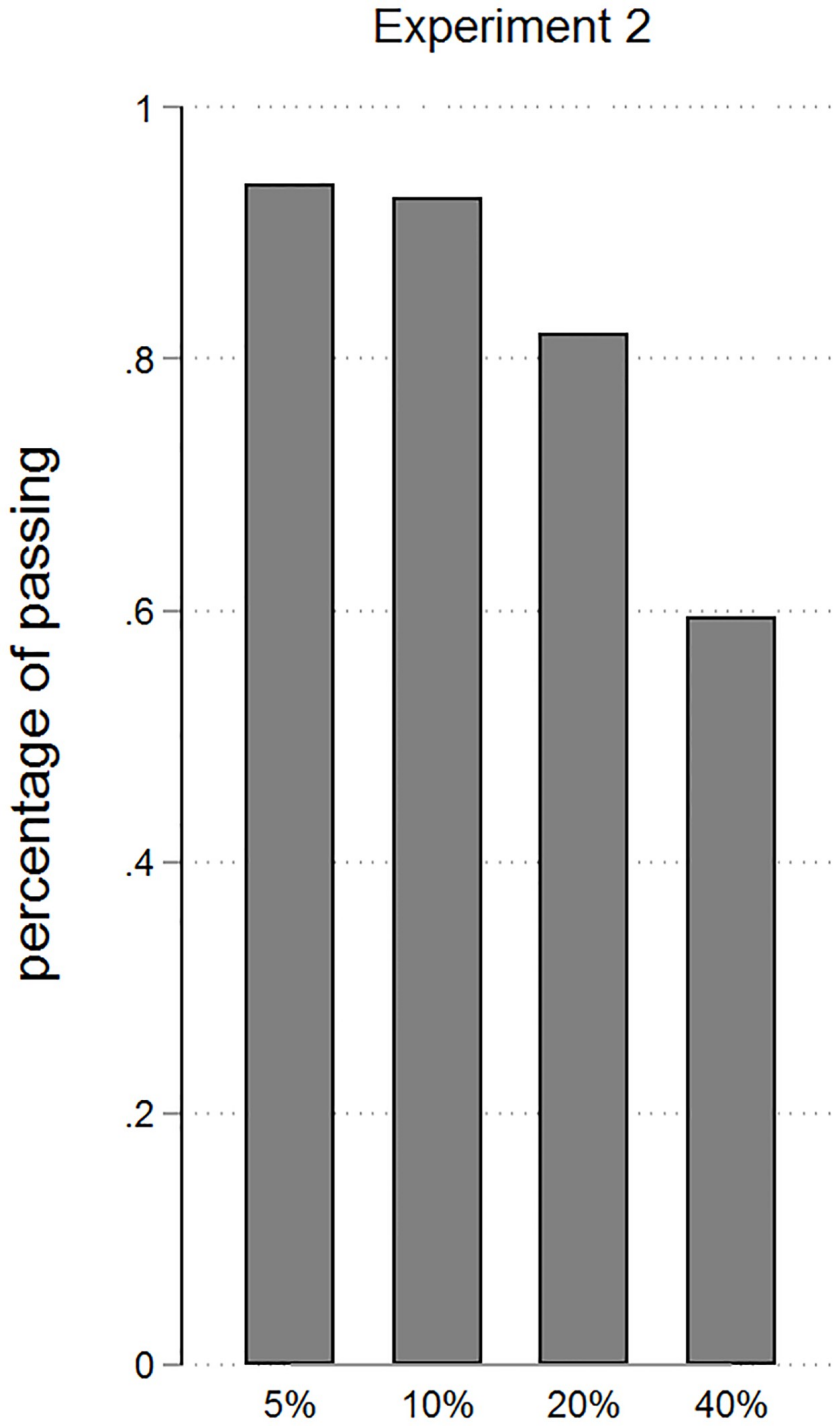
Percentages of passing by stages. Percentages denote the share of participants who decide to pass among all those who have reached a given stage. As can be seen in comparison with Fig 4, percentages are slightly lower than in the treatment “Soccer” but higher than those for “Centipede”.

As shown in Fig 5, we observe conditional percentages of passing of 94%, 93%, 82% and 60% across the 4 stages. This results in a mean number of 1.59 passes with a standard deviation of 0.62. Percentages of passing are slightly lower than in the treatment “Soccer” in experiment 1. Still, values are larger than those for “Centipede” and “Probabilistic” and imply levels of cooperation considerably above those commonly found in similar games. The particular design features and circumstances of this environment make it impossible to draw iron clad conclusions from comparing this experiment to experiment 1. But, taking the results of both experiments together, we posit that the probabilistic design of the centipede game and the frame as a game of soccer are supportive of team reasoning and are likely explanations for cooperation.

## Limitations

The sample in the classroom included students from business administration and economics, which are often found to behave more self-servingly than students from other disciplines. Yet, we also include many students from other disciplines and have an equal distribution of such students across our treatments. Thus, our findings do not suffer from a biased sample. The more representative sample in experiment 2 further supports the validity of our findings.

The difference between probabilistic and non-probabilistic payoffs in experiment 1 may raise the concern, predominantly from psychology, whether participants can adequately translate probabilities into expected payoffs. We consider this concern to be minor due to two reasons. First, a similar distinction has been investigated by [44] in a repeated prisoner’s dilemma without marked differences in the first round (only in subsequent rounds, which are not relevant to our experiment). Second, the probabilities in our experiment serve to simplify understanding rather than complicate the experiment. Doubling the group’s chance to score a goal is intuitive and allows for quick comprehension of the experimental task. We thus would not expect a treatment effect that arises from increased complexity of the instructions.

**Fig 6:**
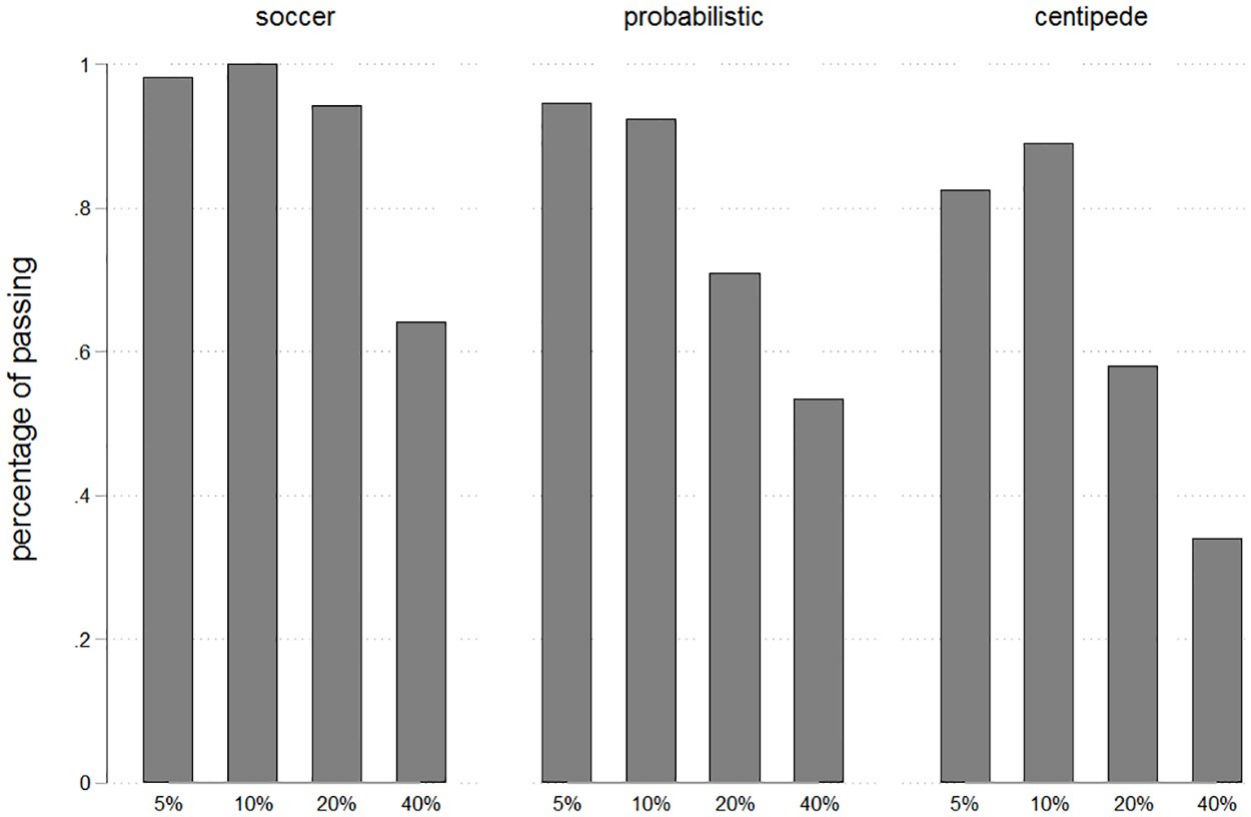
Mean expected percentages of passing by treatment. Percentages denote the share of participants who are expected to pass among all those who will reach the respective stage. As can be seen, percentages are comparable to those in Fig 4.

Our game was played only once, such that participants could not learn across repetitions. Studies with repetition have sometimes brought about reduced cooperation, in particular if a centipede game was repeated with the same partner [19]. One concern could be that our treatment effects may also diminish over time, in particular among players whose partner took earlier. Yet, we do not consider such an effect to be likely. Learning involves an update of beliefs. Players are likely to form beliefs regarding the behavior of their partner and to respond to these beliefs. They might form naïve beliefs in our one-shot game, which induces them to play excessively cooperatively. But, first, our findings are robust to the control for beliefs as shown in the results section of experiment 1. Our treatments have a direct impact on behavior that is not fully explained by their impact on beliefs. Thus, even if beliefs might be updated downward across repetitions, this direct impact is likely to remain. Second, our findings imply that beliefs are not naïve. As shown in Fig 6, expectations are not biased relative to actual behavior. Third, passing is expected slightly too often in “Centipede”. But in “Soccer”, players’ expectations are slightly below the high passing frequencies that are shown in Fig 4. When players can update their beliefs through repetition, they are then likely to expect less passing in “Centipede” and more passing in “Soccer”. This would, if at all, rather suggest that our treatments would exert an even stronger influence if repetitions had been played.

Experiment 1 in the classroom was carried out after experiment 2 and a few participants participated in both. This may have had an impact on behavior. We were aware of this potential influence when running experiment 1 and asked participants to self-report their earlier participation. This was the case for 21 of our 323 participants in experiment 1. In experiment 2, 19 out of 134 subjects had participated in a previous session of experiment 2. Leaving out these subjects does not bring about a noteworthy difference.

Another caveat could be that participants might have communicated and might have seen other people’s smartphone screens. This might induce higher levels of cooperation because reputation might play a role. While we instructed participants to respect the privacy of others, our environment does not guarantee anonymity to a level that can be achieved in the laboratory. In order to address the resulting impact, we asked subjects related questions in the post-experimental questionnaire. They provided self-reported assessments on whether the instructions of their neighbors were identical, differed from theirs or whether they did not know this, the latter option being chosen by 305 of our 323 participants in experiment 1. We also asked whether they had communicated with their neighbors, which was denied by 306 of the 323 participants in experiment 1 and by 124 out of 134 participants in experiment 2. We ran robustness checks and observed that notice of others’ instructions or communication did not impact behavior and that our findings can be replicated for a subsample of participants who did not communicate and who did not know their neighbor’s instructions.

A final limitation relates to our interpretation. Our manipulations may affect behavior not only by increasing the salience of a joint goal. Other explanations may come to mind, for example related to social norms. The frame as a game of soccer may activate a social norm that requests cooperative behavior as, for example, in [45] and [46]. We tested whether our treatments affected the self-reported agreement with the statement: “An individual should subordinate himself/herself to the good of the community”. On a 5-point Likert scale, subjects indicated their approval (1 = totally agree; 5 = totally disagree). In “Soccer”, subjects on average reported a value of 2.49 while in the other treatment and the baseline the average amounted to 2.77. Participants showed significantly higher agreement with this statement in the treatment “Soccer” (Mann-Whitney test, z = 2.66, p<0.01). This reveals that a soccer frame activates a social norm. However, as shown in the regression analysis, this does not impact behavior and inclusion of the variable does not reduce the strength of our treatment effects. Direct evidence in favor of our explanation could be obtained through manipulation checks, identifying whether a joint goal is indeed more salient in our treatments. Such a check would have to assess whether the scoring of a goal in Soccer is more salient as compared to the €128/€32 payoff in “Centipede”. The robust and valid design of such checks is an avenue for future research.

## Discussion

Our lab-in-the-field experiments investigated whether cooperation can be advanced by the choice of a particular experimental design: First, we substituted the individual payoffs commonly reported to subjects by probabilities for achieving a joint goal. Indeed, we observed that this manipulation substantially increased cooperation. Passing was more frequent and persisted even in the final stage. Second, we substituted the neutral instructions of the game with a soccer frame. In this novel and intuitive description as a game of soccer, subjects were put into the role of a soccer player, who can increase the probability of scoring a goal by passing the ball. We observed a further increase in cooperation.

Our findings are best explained by recent theories on team reasoning and the salience of a joint goal. Our probabilistic design made the joint goal more salient. Rather than putting a focus on individual payoffs as in a regular centipede game, it highlights how passing increases the probability of the group’s success. Our soccer frame directs attention towards the soccer goal, the ball, heading in the same direction, the soccer pitch and the continuous passing of the ball as a shared plan. These manipulations are likely to further increase the salience of the joint goal. While we cannot take this as iron-clad evidence for team reasoning, we strongly believe that these manipulations induce the participants to put less focus on the individual advantages and more focus on joint goals. The resulting increase in cooperation can thus be deemed supportive of theories of team reasoning.

Beliefs are one reason for cooperation to increase. Our manipulations can induce subjects to expect the other player to become more cooperative, thus reducing the risk of passing in an early stage. However, our treatments retain a significant influence even when controlling for beliefs and they have a strong impact on passing in the final stage where beliefs do not play a role. This suggests that a theory of team reasoning provides a fertile ground for interpreting our findings. Our manipulations alerted subjects to a joint goal. This is likely to have convinced them to think as a team rather than as an individual.

Existing studies have tried to infer the extent of team reasoning from the game’s payoffs, for example by observing that team reasoning increases if the gains from cooperation are large and the risks are low. Our findings suggest that in addition to such influences, team reasoning also depends on how a game is framed. Salient joint goals can induce subjects to reason as a team rather than as an individual.

We think that our insights are already widely employed in real world situations. Business managers talk at length about their visions, academics dream about projects and publications, and lovers about starting a family. More often than not, they tend to forget about the temptations they will encounter on their way. While their beliefs are likely to be biased, this might secure cooperation. In other areas, our findings might inspire reform. Work morale might be improved more strongly by identifying the organization’s goals rather than by extrinsic incentives aimed at minimizing shirking. Avoiding corruption might be more successful if government agencies shared welfare-enhancing goals rather than by controls that target individual infractions. Conflict resolution is likely to work better if joint goals are emphasized rather than differences between the parties involved in the conflict. These are areas where policymakers and managers can learn from behavioral science and experimental economics.

## Supporting information

S1 File Procedural details and full instructions. (PDF)

## References

[1] Bowles S, Gintis H. A Cooperative Species. Human Reciprocity and its Evolution. Princeton, NJ: Princeton University Press; 2011.

[2] Fehr E, Gächter S. Cooperation and Punishment in Public Goods Experiments. Am Econ Rev. 2000; 90(4): 980–994.

[3] Charness G, Rabin M. Understanding Social Preferences with Simple Tests. Q J Econ. 2002; 117 (3): 817–869.

[4] Fehr E, Falk A. Psychological Foundations of Incentives. Eur Econ Rev. 2002; 46 (4–5): 687–724.

[5] Krockow EM, Colman AM, Pulford BD. Cooperation in Repeated Interactions: A Systematic Review of Centipede Game Experiments, 1992–2016. Eur Rev Soc Psychol. 2016; 27(1): 231–282.

[6] Aumann RJ. Backward induction and common knowledge of rationality. Games Econ Behav. 1995; 8: 6–19.

[7] Aumann RJ. On the Centipede game. Games Econ Behav. 1998; 23, 97–105.

[8] Palacios-Huerta I, Volij O. Field Centipedes. Am Econ Rev. 2009; 99: 1619–1635.

[9] Cox JC, James D. Clocks and Trees: Isomorphic Dutch Auctions and Centipede Games. Econometrica. 2012; 80: 883–903

[10] Rapoport A, Stein WE, Arco JE, Nicholas TE. Equilibrium Play and Adaptive Learning in a Three-Person Centipede Game. Games Econ Behav. 2003; 43(2): 239–65.

[11] Rosenthal RW. Games of perfect information, predatory pricing and the chain-store paradox. J Econ Theory. 1981; 25: 92–100.

[12] Güth W, Ockenfels P, Wendel M. Efficiency by trust in fairness? Multiperiod ultimatum bargaining experiments with an increasing cake. Int J Game Theory. 1993; 22(1): 51–73

[13] Nagel R, Tang FF. Experimental Results on the Centipede Game in Normal Form: An Investigation on Learning. J Math Psychol. 1998; 42: 356–384. 9710555 10.1006/jmps.1998.1225

[14] McKelvey RD, Palfrey TR. An Experimental Study of the Centipede Game. Econometrica. 1992; 60: 803–836.

[15] Brandts J, Charness G. The Strategy Versus the Direct-Response Method: A First Survey of Experimental Comparisons. Exp Econ. 2011; 14(3): 375–398.

[16] Dufwenberg M, Kirchsteiger G. A theory of sequential reciprocity. Games Econ Behav. 2004; 47: 268–298.

[17] Levitt SD, List JA, Sadoff SE. Checkmate: Exploring Backward Induction among Chess Players. Am Econ Rev. 2011; 101: 975–990.

[18] López-Pérez R. Aversion to norm-breaking: A model. Games Econ Behav. 2008; 64: 237–267.

[19] Krockow EM, Colman AM, Pulford BD. Exploring cooperation and competition in the Centipede game through verbal protocol analysis. Europ J Soc Psychol. 2016; 46(6): 746–761.

[20] Pulford BD, Krockow EM, Colman AM, Lawrence CL. Social value induction and cooperation in the Centipede game. PLoS One. 2016; 11(3): 1–21. 10.1371/journal.pone.0152352 PMC4806875 27010385

[21] Pulford BD, Colman AM, Lawrence CL, Krockow EM. Reasons for Cooperating in Repeated Interactions: Social Value Orientations, Fuzzy Traces, Reciprocity, and Activity Bias. Decision. 2017; 4(2): 102–122.

[22] Bacharach M. Interactive Team Reasoning: A Contribution to the Theory of Cooperation. Res Econ. 1999; 53(2): 117–147.

[23] Bacharach M, Gold N, Sugden R. Beyond Individual Choice. Princeton NJ: Princeton Univ. Press; 2006.

[24] Sugden R. The Logic of Team Reasoning. Philos Explor. 2003; 6 (3): 165–181.

[25] Bardsley N, Mehta J, Starmer C, Sugden R. Explaining Focal Points: Cognitive Hierarchy Theory versus Team Reasoning. Econ J. 2009; 120: 40–79.

[26] Tan J, Zizzo D. Groups, Cooperation and Conflict in Games. J Socio Econ. 2008; 37 (1): 1–17.

[27] Sugden R. Mutual advantage, conventions and team reasoning. Int Rev Econ. 2011; 58, 9–20.

[28] Capraro V. A Model of Human Cooperation in Social Dilemmas. PLoS One. 2013; 8, e72427 10.1371/journal.pone.0072427 24009679 10.1371/journal.pone.0072427 PMC3756993

[29] Barcelo H, Capraro V. Group Size Effect on Cooperation in One-Shot Social Dilemmas. Scientific Reports. 2015: 5, 7937 10.1038/srep07937 25605124 10.1038/srep07937 PMC4300455

[30] Capraro V, Polukarov M, Venanzi M, Jennings NR. Cooperative equilibria beyond social dilemmas: Pareto solvable games. 2015; https://arxiv.org/abs/1509.07599.

[31] Capraro V, Venanzi M, Polukarov M, Jennings NR. Cooperative equilibria in iterated social dilemmas. Proceedings of the 6th International Symposium on Algorithmic Game Theory, Lecture Notes in Computer Science. 2013; 8146: 146–158.

[32] Tuomela R. Cooperation: A Philosophical Study. Dordrecht and Boston: Kluwer Acad Publ; 2000.

[33] Hakli R, Millera K, Tuomela R. Two Kinds of We-reasoning. Econ Phil. 2010; 26 (3): 291–320.

[34] Hindriks F. Team reasoning and group identification. Ration Soc. 2012; 24 (2): 198–220.

[35] Bratman ME. Shared Agency: A Planning Theory of Acting Together. Oxford UK: Oxford University Press; 2014.

[36] Tomasello M. A natural history of human thinking Cambridge Mass: Harvard Univ. Press; 2014.

[37] Misyak JB, Chater N. Virtual bargaining: a theory of social decision-making. Philos Trans Royal So B. 2014; 369: 1–9. 10.1098/rstb.2013.0487 PMC4186239 25267828

[38] Colman AM, Gold N. Team reasoning: Solving the puzzle of coordination. Psychon Bull Rev. 2017: 1–14. 29101730 10.3758/s13423-017-1399-0

[39] Krockow EM, Takezawa M, Pulford BD, Colman AM, Kita T. Cooperation and trust in Japanese and British samples: Evidence from incomplete information games. Int Perspect Psychol. 2017; 6(4): 227–245.

[40] Deb S, Dey D. Spatial Modeling of Shot Conversion in Soccer to Single out Goalscoring Ability; 2017. https://arxiv.org/abs/1702.05662.

[41] Giamattei M, Lambsdorff J. classEx—an Online Software for Classroom Experiments; 2015. Passau Working Paper.

[42] Le Coq C, Tremewan J, Wagner AK. On the effects of group identity in strategic environments. Euro Econ Rev. 2015; 76: 239–252.

[43] Lambsdorff J, Giamattei M, Werner K. How Fragile is Conditional Cooperation? A Field Experiment with Smartphones during the 2014 Soccer World Cup. J Behav Dec Making. 2017; 30(2): 492–501.

[44] Bereby-Meyer Y, Roth AE. The Speed of Learning in Noisy Games: Partial Reinforcement and the Sustainability of Cooperation. Am Econ Rev. 2006; 96 (4): 1029–1042.

[45] Liberman V, Samuels SM, Ross L. The name of the game: predictive power of reputations versus situational labels in determining prisoner’s dilemma game moves. Pers Soc Psychol Bull. 2004; 30 (9): 1175–1185. 15359020 10.1177/0146167204264004

[46] Kay AC, Wheeler SC, Bargh JA, Ross L. Material priming: The influence of mundane physical objects on situational construal and competitive behavioral choice. Organ Behav Hum Decis Process. 2004; 95 (1): 83–96.

