# Supplementary material for: Team reasoning—Experimental evidence on cooperation from centipede games
Source: PLoS One. 2018 Nov 28;13(11):e0206666. doi: 10.1371/journal.pone.0206666 (PMC6261539; doi:10.1371/journal.pone.0206666)
Supplement: S1 File — (PDF) [file pone.0206666.s001.pdf]

Team Reasoning – Experimental Evidence on Cooperation from Centipede Games

Procedural Details and Full Instructions

Johann Graf Lambsdorff, Marcus Giamattei, Katharina Werner, and Manuel Schubert

The full set of instructions can be found in this document.

All collected data can be found at https://dx.doi.org/10.23663/x2574.

Experiment 1 2

Procedural Details 2

Public Announcements in Class (English Translation) 3

Written Instructions on Flyers 5

On-Screen Instructions 7

Original Written Instructions in German 11

Experiment 2 15

Procedural Details 15

Public Announcements (English Translation) 16

Written Instructions on Flyers 17

On-Screen Instructions 20

Sample Screens (original German version) 24

Public Announcements (German version) 25

Written Instructions on Flyers (German version) 27

Instructions on the smartphone (German version) 30

Experiment 1

Fig S1 shows the classroom during experiment 1, December 10, 2014.

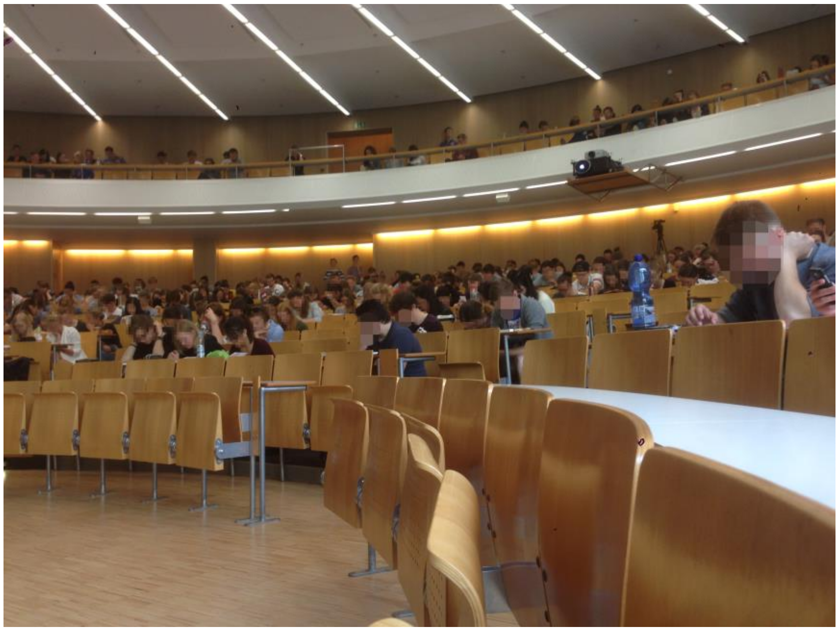

Fig S1. Classroom during experiment 1

Procedural Details

All instructions were in German. Below is the English translation. The instructions consist of public announcements, written instructions on flyers, which were distributed to participants, and instructions on the screen of the mobile device.

We used the strategy method combined with certain features of the game method: Subjects decided independently of the other player, as in a strategy method design, to keep data traffic minimal. At the same time, they were guided through the experiment step-by-step as if they were interacting with another player. In this sequential design, if they decided to take at a certain stage, they did not have to make any further decision, as if they were playing game method. After each decision, subjects were asked to state their beliefs whether the other player would take or pass in the subsequent stage. Matching of participants into pairs of players took place at the end of the

experiment, prior to drawing the winning pairs. This design choice eliminates problems with participants dropping out during the course of the experiment.

Public Announcements in Class (English Translation)

Welcome to the lecture in microeconomics. Today, I will leave the lecture earlier. After I have left, you can participate in a classroom game ran by my colleague Graf Lambsdorff. Participation is optional and is not related to the lecture. All smartphone users can participate and earn a total amount of more than 1500€. The game is used to collect data for research purposes. More detailed instructions on the game can be found on the flyer in front of you. The exact course of the game is explained on the flyer and illustrated in a graph. (This part of the announcement was made at 10:15 a.m. by Michael Grimm, professor in microeconomics, the following part of the announcement was made by Johann Graf Lambsdorff at 11 a.m.).

Welcome to today's classroom game. All smartphone users can participate and earn a total amount of more than 1500€. We are a team of the University of Passau and want to collect data for research purposes. More detailed instructions on the game can be found on the flyer in front of you. Before starting the game, I am now going to explain the game once more.

Once the game starts, you will be assigned to a group and a role. Your group consists of you and another player in the classroom who will be randomly selected by the computer. Instructions on your smartphone will guide you through the game step by step. Please always read the texts on your smartphone carefully before making a decision. You will have sufficient time to do so. The payoff you can receive depends on your own decisions and those of the other randomly selected player in the classroom. The exact course of the game is explained on the flyer and illustrated in a graph. In case you have not read the flyer yet, you will get some short time to do so in a moment. After the game, you will be asked to answer some questions for statistical purposes. The computer will then randomly select ten groups among all participants that will receive the payoffs according

to the rules stated on the flyer. Each player receives a player number which will be displayed on the smartphone at the end of the game. Don’t lose this player number. Right after the game, we will read out the player numbers that have won. If you have won you will receive the respective amount immediately and in cash upon presentation of your smartphone with the player number – depending on your role either upstairs in the hallway or down here behind the side exit. It is stated on the flyer where you will receive your payoff.

All of your decisions and data are anonymous. They can neither be observed by the other player or the players of other groups nor by us. The persons distributing the payoffs do not know the game and cannot infer your behavior from the amount you receive. Please respect other people’s privacy and do not look at their smartphones. Please read the flyer carefully now because you will have to answer comprehension questions at the beginning of the game and you will only be able to participate after answering them correctly. I will give you 4 more minutes now for reading. Then I will start the game (After 5 minutes the last part of the announcement was made).

When I start the game in a moment, you will have 5-10 minutes to complete the game. This is a long time and you can think about your decisions without any hurry. From my point of view, we can start now.

Written Instructions on Flyers

The following instructions for treatment "Soccer" were printed and given to participants upfront. For the treatments "Probabilistic" the words and expression were substituted as shown in Table S1.

Table S1. Different wordings for the Soccer and the Probabilistic Frame.

"Soccer"	"Probabilistic"
Team	Group
Position	Role
Scorer	Player who takes
Teammate	Other player
Right wing	Player A
Left wing	Player B
Shoot	Take
Ball	Decision
Goal	Success

We are a team of the University of Passau and want to collect data for research purposes. The classroom game will be played in the lecture in microeconomics and will take about 10 minutes. All smartphone users can participate and earn a total amount of more than 1500€. If you want to participate, please read the following instructions carefully.

Before the game – good preparation. When the game starts, open the webpage classEx.uni-passau.de/start. There you will be assigned to a team and a position. Your team consists of you and another randomly selected player. Your position is either right wing or left wing.

Start – The attack on the goal begins. Right wing (R) and left wing (L) are approaching the opponent's goal. The goal scorer receives 160€, the team mate 40€ [Centipede: The player who takes earns a higher payoff than the other player]. [Figs 1-3 from the main text was shown, depending on the treatment; the following description is for "Soccer"; Table S1 details changes for "Probabilistic". For "Centipede" probabilities must be further exchanged for non-probabilistic payoffs.]

- Right wing has the ball and can either SHOOT or PASS. If he SHOOTS he will score a goal with a probability of 5%. If he PASSES the ball goes to left wing.

- If left wing receives the ball he can also decide whether to SHOOT or PASS. With SHOOT he will score a goal with a probability of 10%. If he PASSES the ball goes back to right wing.
- If right wing gets the ball back he can again SHOOT or PASS. With SHOOT he will score a goal with a probability of 20%. If he PASSES the ball goes to left wing again.
- If left wing gets the ball again he can choose between SHOOT or PASS one last time. With SHOOT he will score a goal with a probability of 40%. If he PASSES the ball goes back to right wing who now is in an optimal position and will score a goal with a probability of 80%.

After the game some questions for statistical purposes are to be answered.

Finish – Have you won? Randomly, 10 teams will be selected among all participants after the game has finished. These teams will receive the goal bonus if they have scored a goal. The goal scorer receives 160€, the team mate 40€. Each player receives a player number which will be displayed on the smartphone at the end of the game. Don't lose this player number. Right after the game, we will read out the player numbers that have won. If you have won, you will receive the respective amount immediately and in cash upon presentation of your smartphone with the player number. Rights wings will receive their payoff upstairs in the hallway, lefts wings will receive it down here, behind the side exit (In the field the instruction was changed to: If you have won you will receive the respective amount immediately and in cash upon presentation of your smartphone with the player number at our information desk). All of your decisions and information are anonymous. Your decisions can neither be observed by the players of other teams nor by us. Nor will you ever learn who your team mate is. Please respect other people's privacy and do not look at their smartphones.

On-Screen Instructions

The on screen instructions can be found in Tables S2-S5. Each screen displayed only one question. Again terms were changed according to Table S1 for the treatments “Probabilistic” and “Centipede”.

Table S2. On-screen instructions for both players (treatment “Soccer”).

Number	Question Text	Type of Question	Options	Explanation
1	If you SHOOT, you have the chance to earn…	Single Choice	10€ (2)	Read the flyer carefully. If you want to start the game, please answer two comprehension questions on the game. Then you can start!
			40€ (2)	
			80€ (2)	
			160€ (3)	
2	If you SHOOT, you have the chance to earn…	Single Choice	10€ (2)	You provided the wrong answer. Please try again.
			40€ (2)	
			80€ (2)	
			160€ (3)	
3	If you PASS, …	Single Choice	the chance of scoring a goal decreases (4).	You answered the first question correctly. Please answer another question, then you can start.
			the chance of scoring a goal remains identical. (4).	
			the chance of scoring a goal increases (5).	
4	If you PASS, …	Single Choice	the chance of scoring a goal decreases (4).	You provided the wrong answer. Please try again.
			the chance of scoring a goal remains identical. (4).	
			the chance of scoring a goal increases (5).	
Continue with question 5 in Table S3 for right wing and in Table S4 for left wing.				

Notes: The numbers in brackets in column “Options” denote the following question number if the participant decided in favor of that option).

Table S3. On-screen instructions for right wing player (treatment "Soccer").

Num-ber	Question Text	Type of Question	Options	Explanation
5	You have the ball and you can SHOOT or PASS. If you SHOOT you score a goal with a probability of 5%. If you PASS the ball goes to left wing and he can decide. What do you do?	Single Choice	I SHOOT (6)<br>I PASS (7)	You answered the questions correctly. The game will start now.
6	If you had passed, do you think left wing would have SHOT at his 10% chance for a goal or would he have PASSED back to you?	Single Choice	Left wing would have SHOT (11)<br>Left wing would have PASSED (11)	You shot.
7	Do you think left wing will SHOOT at his 10% chance for a goal or will he PASS back to you?	Single Choice	Left wing will SHOOT (8)<br>Left wing will PASS (8)	You passed.
8	Assume that left wing PASSED at his chance for a goal of 10%. You can now SHOOT or PASS the ball. If you SHOOT you score a goal with a probability of 20%. If you PASS the ball goes to left wing and he can decide. What do you do?	Single Choice	I SHOOT (9)<br>I PASS (10)	Left wing is deciding whether to shoot or pass.
9	If you had passed, do you think, left wing would have SHOT at his 40% chance for a goal or would he have PASSED back to you?	Single Choice	Left wing would have SHOT (11)<br>Left wing would have PASSED (11)	You shot.
10	Do you think left wing will SHOOT at his 40% chance for a goal or will he PASS back to you?	Single Choice	Left wing will SHOOT (11)<br>Left wing will PASS (11)	You passed. Left wing is deciding whether to if he shoots or passes. If left wing passes, you shoot automatically.
Continue with question 11 in Table S5.				

Notes: The numbers in brackets in column "Options" denote the following question number if the participant decided in favor of that option).

Table S4. On-screen instructions for left wing player (treatment "Soccer").

Num-ber	Question Text	Type of Question	Options	Explanation
5	Right wing has the ball and he can SHOOT or PASS. If he SHOOTS he scores a goal with a probability of 5%. If he PASSES the ball goes to you and you can decide. What do you think, right wing will do?	Single Choice	Right wing will SHOOT (6)	You answered the questions correctly. The game will start now.
			Right wing will PASS (6)	
6	Assume that right wing PASSED at his chance for a goal of 5%. You can now SHOOT or PASS the ball. If you SHOOT you score a goal with a probability of 10%. If you PASS the ball goes to right wing and he can decide. What do you do?	Single Choice	I SHOOT (7)	
			I PASS (8)	
7	If you had passed, do you think right wing would have SHOT at his 20% chance for a goal or would he have PASSED back to you?	Single Choice	Right wing would have SHOT (11)	You shot.
			Right wing would have PASSED (11)	
8	Do you think, right wing will SHOOT at his 20% chance for a goal or will he PASS back to you?	Single Choice	Right wing will SHOOT (9)	You passed.
			Right wing will PASS (9)	
9	Assume that right wing PASSED at his chance for a goal of 20%. You can now SHOOT or PASS the ball. If you SHOOT you score a goal with a probability of 40%. If you PASS the ball goes back to right wing and he SHOOTS automatically. Thereby he scores a goal with a probability of 80%. What do you do?	Single Choice	I SHOOT (11)	Right wing is deciding whether to shoot or to pass.
			I PASS (11)	
Continue with question 11 in Table S5.				

Notes: The numbers in brackets in column "Options" denote the following question number if the participant decided in favor of that option).

Table S5. Post-experimental questionnaire for both players (treatment "Soccer").

Num-ber	Question Text	Type of Question	Options
11		Single Choice	I am male.
			I am female
12	I study...	Single Choice	Business Administration / Economics
			Governance and Public Policy
			Teaching
			International Cultural and Business Studies
			Something else
13		Single Choice	I am less than 20 years old.
			I am between 20 and 24 years old.
			I am between 25 and 30 years old.
			I am more than 30 years old.
14		Single Choice	The people sitting next to me had the same instructions as me.
			The people sitting next to me partly had other instructions than me.
			I did not take notice of the instructions of the people sitting next to me.
15	Did you discuss your decision with other smartphones users (participants)?	Single Choice	Yes
			No
16	Did you participate at the game Shoot or Pass in the lecture hall or at the public viewing event during the soccer world cup in July 2014?	Single Choice	Yes
			No
17	In general, I tend to…	Likert Scale	1 = avoid risks
			5 = take risks.
18	"An individual should subordinate himself/herself to the good of the community." With this statement…	Likert Scale	1 = I totally agree.
			5 = I totally disagree.
19	How are you feeling? At the moment I am…	Likert Scale	1 = relaxed / calm
			5= aroused / tense

Original Written Instructions in German

Original instructions in German are shown in Figs S2 – S4. Leaflets were folded such that reading starts on the right hand side of the upper page and ends on that pages left hand side.

Im Anschluss sind noch ein paar Fragen für statistische Zwecke zu beantworten.

3. Ende – Hast Du gewonnen?

Per Zufallsverfahren werden nach Spielende unter allen Teilnehmern 10 Gruppen ausgewählt. Diese erhalten die aus dem Spiel resultierenden Auszahlungen.

Jeder Spieler erhält eine Spielernummer, die am Ende des Spiels auf dem Smartphone angezeigt wird. Verliere diese Nummer nicht. Sofort nach Spielende lesen wir die Spielernummern vor, die gewonnen haben. Wenn Du gewonnen hast, wird Dir gegen Vorlage Deiner Spielernummer sofort der entsprechende Betrag bar ausbezahlt. Die Spieler A erhalten die Auszahlung oben im Foyer, die Spieler B erhalten sie unten, hinter dem Nebenausgang.

Alle Deine Entscheidungen und Informationen bleiben anonym. Deine Entscheidungen können weder von Spielern anderer Gruppen noch von uns beobachtet werden. Auch erfährst Du nicht, mit wem du gespielt hast. Bitte respektiere die Privatsphäre anderer und schaue nicht auf deren Smartphones.

HÖRSAALSPIEL

Wir sind ein Team der Universität Passau und erheben Daten für Forschungszwecke. Das Hörsaalspiel wird in der Vorlesung Makroökonomik durchgeführt und dauert ca. 10 Minuten. Alle Inhaber eines Smartphones können teilnehmen und insgesamt über 1500€ verdienen. Wenn Du mitmachen möchtest, lies die folgenden Anweisungen aufmerksam durch.

1. Vor Spielbeginn – Gut vorbereiten

Öffne zum Beginn des Spiels die Seite

classEx.uni-passau.de/anfang

Dort wirst Du einer Gruppe und einer Rolle zugeteilt. Deine Gruppe besteht aus Dir und einem zufällig ausgewählten anderen Spieler. Deine Rolle ist entweder Spieler A oder Spieler B.

2. Start – Das Spiel beginnt

Die Spieler A und B können unterschiedliche Auszahlungen erzielen. Der Spieler, der NIMMT, erhält eine höhere Auszahlung als der andere Spieler.

- Spieler A hat anfangs die Entscheidung und kann NEHMEN oder PASSEN. Falls er NIMMT, erhält er 8€ und B erhält 2€. Falls er PASST, geht die Entscheidung an B.
- Wenn B die Entscheidung bekommt, steht er ebenfalls vor der Entscheidung NEHMEN oder PASSEN. Mit NEHMEN erhält er 16€ und A erhält 4€. Falls er PASST, geht die Entscheidung an A zurück.
- Wenn A die Entscheidung zurückbekommt, kann er erneut NEHMEN oder PASSEN. Falls er NIMMT, erhält er 32€ und B erhält 8€. Falls er PASST, geht die Entscheidung erneut an B.
- Wenn B die Entscheidung wieder bekommt, wählt er ein letztes Mal zwischen NEHMEN oder PASSEN. Falls er NIMMT, erhält er 64€ und A erhält 16€. Falls er PASST, wird automatisch A NEHMEN und damit 128€ erhalten. B erhält dann 32€.

Der Spielablauf wird durch folgende Grafik illustriert.

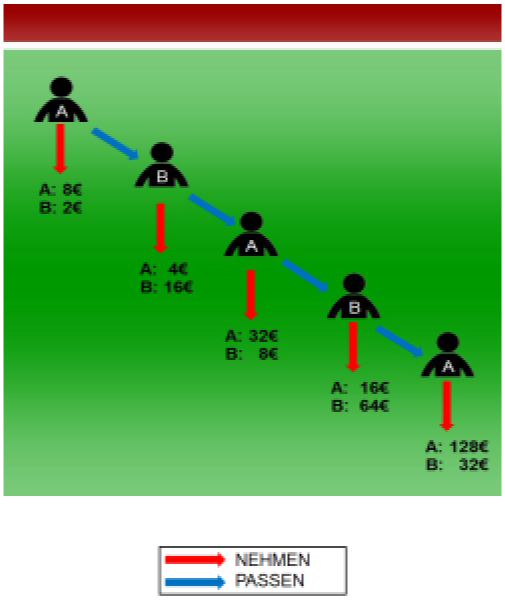

Fig S2. Original instructions for treatment "Centipede".

Im Anschluss sind noch ein paar Fragen für statistische Zwecke zu beantworten.

3. Ende – Hast Du gewonnen?

Per Zufallsverfahren werden nach Spielende unter allen Teilnehmern 10 Gruppen ausgewählt. Diese erhalten die Auszahlung, wenn Sie erfolgreich waren: Der Spieler, der GENOMMEN hat, erhält 160€, der andere 40€.

Jeder Spieler erhält eine Spielernummer, die am Ende des Spiels auf dem Smartphone angezeigt wird. Verliere diese Nummer nicht. Sofort nach Spielende lesen wir die Spielernummern vor, die gewonnen haben. Wenn Du gewonnen hast, wird Dir gegen Vorlage Deiner Spielernummer sofort der entsprechende Betrag bar ausbezahlt. Die Spieler A erhalten die Auszahlung oben im Foyer, die Spieler B erhalten sie unten, hinter dem Nebenausgang.

Alle Deine Entscheidungen und Informationen bleiben anonym. Deine Entscheidungen können weder von Spielern anderer Gruppen noch von uns beobachtet werden. Auch erfährst Du nicht, mit wem du gespielt hast. Bitte respektiere die Privatsphäre anderer und schaue nicht auf deren Smartphones.

HÖRSAALSPIEL

Wir sind ein Team der Universität Passau und erheben Daten für Forschungszwecke. Das Hörsaalspiel wird in der Vorlesung Mikroökonomik durchgeführt und dauert ca. 10 Minuten. Alle Inhaber eines Smartphones können teilnehmen und insgesamt über 1500€ verdienen. Wenn Du mitmachen möchtest, lies die folgenden Anweisungen aufmerksam durch.

1. Vor Spielbeginn – Gut vorbereiten

Öffne zum Beginn des Spiels die Seite

classEx.uni-passau.de/beginn

Dort wirst Du einer Gruppe und einer Rolle zugeteilt. Deine Gruppe besteht aus Dir und einem zufällig ausgewählten anderen Spieler. Deine Rolle ist entweder Spieler A oder Spieler B.

2. Start – Das Spiel beginnt

Die Spieler A und B können mit unterschiedlichen Wahrscheinlichkeiten einen Erfolg erzielen. Der Spieler, der NIMMT, erhält 160€. Der andere Spieler erhält 40€.

- Spieler A hat anfangs die Entscheidung und kann NEHMEN oder PASSEN. Falls er NIMMT, erzielt er einen Erfolg mit 5% Wahrscheinlichkeit. Falls er PASST, geht die Entscheidung an Spieler B.
- Wenn B die Entscheidung bekommt, steht er ebenfalls vor der Entscheidung NEHMEN oder PASSEN. Falls er NIMMT, erzielt er einen Erfolg mit 10% Wahrscheinlichkeit. Falls er PASST, geht die Entscheidung an A zurück.
- Wenn A die Entscheidung zurückbekommt, kann er erneut NEHMEN oder PASSEN. Falls er NIMMT, erzielt er einen Erfolg mit 20% Wahrscheinlichkeit. Falls er PASST, geht die Entscheidung erneut an B.
- Wenn B die Entscheidung wieder bekommt, wählt er ein letztes Mal zwischen NEHMEN oder PASSEN. Falls er NIMMT, erzielt er einen Erfolg mit 40% Wahrscheinlichkeit. Falls er PASST, wird A automatisch NEHMEN und erzielt einen Erfolg mit 80% Wahrscheinlichkeit.

Der Spielablauf wird durch folgende Grafik illustriert.

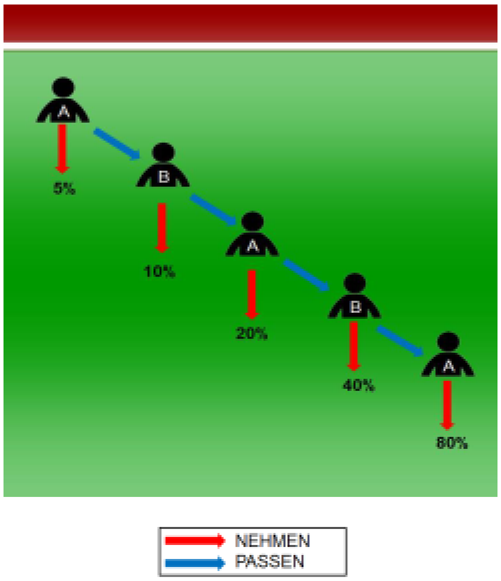

Fig S3. Original instructions for treatment "Probabilistic".

Im Anschluss sind noch ein paar Fragen für statistische Zwecke zu beantworten.

3. Ende – Hast Du gewonnen?

Per Zufallsverfahren werden nach Spielende unter allen Teilnehmern 10 Mannschaften ausgewählt. Diese erhalten die Torprämien ausbezahlt, sofern sie ein Tor geschossen haben. Der Torschütze erhält 160€, der Mitspieler 40€.

Jeder Spieler erhält eine Spielernummer, die am Ende des Spiels auf dem Smartphone angezeigt wird. Verliere diese Nummer nicht. Sofort nach Spielende lesen wir die Spielernummern vor, die gewonnen haben. Wenn Du gewonnen hast, wird Dir gegen Vorlage Deiner Spielernummer sofort der entsprechende Betrag bar ausbezahlt. Rechtsaußen erhalten die Auszahlung oben im Foyer, Linksaußen erhalten sie unten, hinter dem Nebenausgang.

Alle Deine Entscheidungen und Informationen bleiben anonym. Deine Entscheidungen können weder von Spielern anderer Mannschaften noch von uns beobachtet werden. Auch erfährst Du nicht, wer Dein Mitspieler ist. Bitte respektiere die Privatsphäre anderer und schaue nicht auf deren Smartphones.

HÖRSAALSPIEL

Wir sind ein Team der Universität Passau und erheben Daten für Forschungszwecke. Das Hörsaalspiel wird in der Vorlesung Mikroökonomik durchgeführt und dauert ca. 10 Minuten. Alle Inhaber eines Smartphones können teilnehmen und insgesamt über 1500€ verdienen. Wenn Du mitmachen möchtest, lies die folgenden Anweisungen aufmerksam durch.

1. Vor Spielbeginn – Gut vorbereiten

Öffne zum Beginn des Spiels die Seite

classEx.uni-passau.de/start

Dort wirst Du einer Mannschaft und einer Position zugeteilt. Deine Mannschaft besteht aus Dir und einem zufällig ausgewählten Mitspieler. Deine Position ist entweder Rechtsaußen oder Linksaußen.

2. Start – Der Angriff beginnt

Rechtsaußen (R) und Linksaußen (L) laufen auf das gegnerische Tor zu. Der Torschütze erhält 160€, der Mitspieler 40€.

- Rechtsaußen hat den Ball und kann SCHIESSEN oder PASSEN. Falls er SCHIESST, erzielt er ein Tor mit 5% Wahrscheinlichkeit. Falls er PASST, geht der Ball an Linksaußen.
- Wenn Linksaußen den Ball bekommt, steht er ebenfalls vor der Entscheidung SCHIESSEN oder PASSEN. Mit SCHIESSEN erzielt er ein Tor mit 10% Wahrscheinlichkeit. Falls er PASST, geht der Ball an Rechtsaußen zurück.
- Wenn Rechtsaußen den Ball zurückbekommt, kann er erneut SCHIESSEN oder PASSEN. Falls er SCHIESST, erzielt er ein Tor mit 20% Wahrscheinlichkeit. Falls er PASST, geht der Ball erneut an Linksaußen.
- Wenn Linksaußen den Ball wieder bekommt, wählt er ein letztes Mal zwischen SCHIESSEN oder PASSEN. Falls er SCHIESST, erzielt er ein Tor mit 40% Wahrscheinlichkeit. Falls er PASST, geht der Ball an Rechtsaußen zurück, der optimal steht und ein Tor mit 80% Wahrscheinlichkeit erzielt.

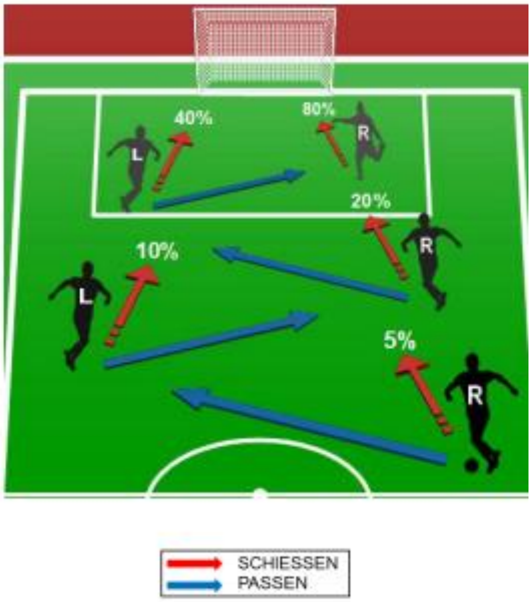

Fig S4. Original instructions for treatment "Soccer".

Experiment 2

Procedural Details

The second experiment was carried out in two sessions at public viewing events, i.e. in beer gardens where large-screen live broadcasts of the respective soccer match were shown. The first session was run during the quarterfinal match Germany against France with approximately 1200 fans in a Bavarian beer garden (see Fig S5). The second session was run during the final Germany against Argentine in a public viewing event with approximately 1800 visitors in a Bavarian brewery (see Fig S6). Methods employed in the field are explained in a movie, to be found at https://www.youtube.com/watch?v=C4ajsNmy9Fk.

All instructions were in German. Below is the English translation, followed by the original German version. The instructions consisted of public announcements, written instructions on flyers which were distributed to participants as well as on-screen instructions. Terms in brackets were different between the quarter-final and the final. Some sample screens are shown in the original German version together with the full on-screen instructions as displayed on the mobile device.

Again, we used the strategy method combined with certain features of the game method (see first environment). In total, we collected 379 observations in the field. 245 participants were additionally provided with the information that they were playing for Germany or for the opponent team (France or Argentina). Findings from these conditions are reported in (43). Here, we only process data from the group that was given no such information (91 observations from the quarterfinal and 43 from the final). From the 379 participants, 25 teams were randomly chosen with 11 scoring a goal, such that €2,200 were paid out. Of these 25 teams, eight were drawn from the 134 subjects whose data is reported in this paper.

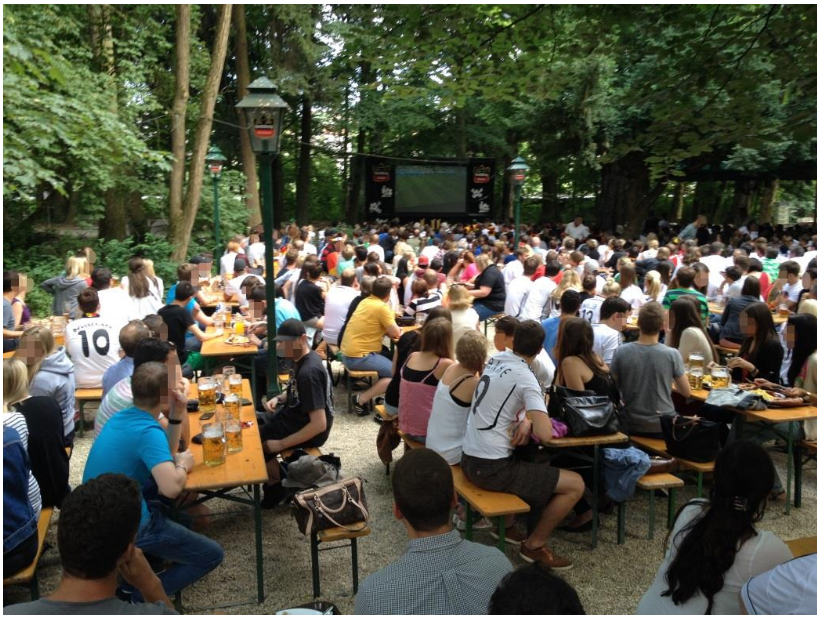

Fig S5. Environment of experiment 2 during the quarterfinal

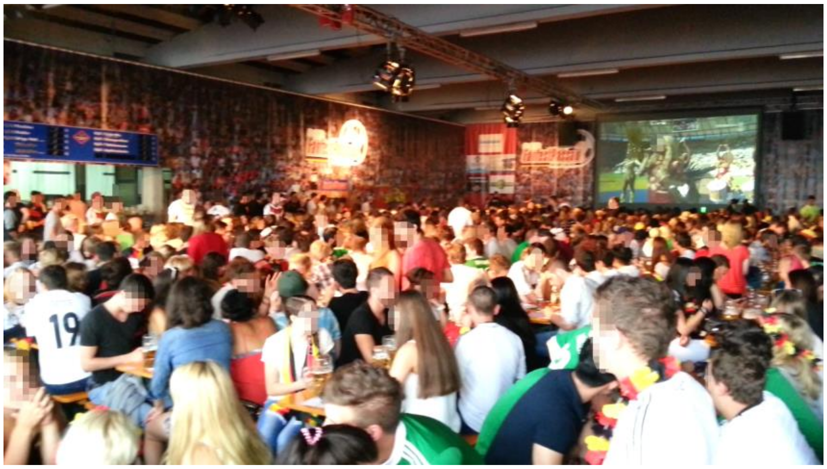

Fig S6. Environment of experiment 2 during the final

Public Announcements (English Translation)

Hi everybody and welcome to the game SHOOT or PASS. If you have questions concerning the game, you can approach our assistants in the yellow t-shirts at any time. In order to participate in the game, you should read the flyer carefully and open the website stated on the flyer.

Everybody can participate and win 160€ as a goal scorer or 40€ as a team mate. [We have already played that game twice in another location and have paid out a total of 2560€ there.] Today, a total amount of 3000€ may be paid out. The game will take approximately 5 minutes. Participation pays!

As soon as the game starts, all of you will be randomly assigned to a group, consisting of you and another randomly chosen player here at the public viewing event in this beer garden. Your position will either be right wing or left wing. Several times, you will have to decide between SHOOT and PASS. With SHOOT you have the chance to win the high bonus of 160€. With PASS your group's chance of scoring a goal increases.

The game will start shortly before the kick-off of the quarterfinal Germany vs. France [final Germany vs. Argentina]. You have time until the 30th minute of the quarterfinal [final]. Then we will stop the smartphone game.

An identification code will be displayed on your smartphone. In the half-time break, I will read out the winners' identification codes. The winners will receive their goal bonus immediately in cash at our information desk. Thank you!

Written Instructions on Flyers

SHOOT or PASS

We are a team of the University of Passau and want to collect data for research purposes. SHOOT or PASS starts shortly before the kick-off and runs until the 30th minute of today's Soccer World Cup game. You can make decisions with your smartphone, shoot a goal and earn money. Everyone can participate and earn 160€ as a goal scorer or 40€ as a team mate. If you want to participate, please read the following instructions carefully.

1. **Before the game – good preparation.** Please open the webpage classEx.uni-passau.de/wm shortly before the kick-off of the game. *Additionally, a QR code was shown.* There you will be

assigned to a team and a position. Your team consists of you and another randomly selected player. Your position is either right wing or left wing.

2. **Start – The attack on the goal begins.** Right wing (R) and left wing (L) are approaching the opponent's goal. *Fig 1 from the main text was shown.*

- Right wing has the ball and can either SHOOT or PASS. If he SHOOTS he will score a goal with a probability of 5%. If he PASSES the ball goes to left wing.
- If left wing receives the ball he can also decide whether to SHOOT or PASS. With SHOOT he will score a goal with a probability of 10%. If he PASSES the ball goes back to right wing.
- If right wing gets the ball back he can again SHOOT or PASS. If he decides to SHOOT he will score a goal with a probability of 20%. If he PASSES the ball goes to left wing again.
- If left wing gets the ball again he can choose one last time between SHOOT and PASS. With SHOOT he will score a goal with a probability of 40%. If he PASSES the ball goes back to right wing who now is in an optimal position and will score a goal with a probability of 80%.

After the game, some questions for statistical purposes are to be answered.

3. **Finish – Have you won?** After the game has finished, 10 [15] teams will be randomly selected among all participants. These teams will receive the goal bonus if they have scored a goal. The goal scorer receives 160€, the team mate 40€.

Each player receives an identification code which will be displayed on the smartphone at the end of the game. Don't lose this identification code. In the half-time break, we will read out the identification codes that have won. If you have won, the respective amount will be paid out immediately in cash upon presentation of your smartphone with the identification code at our information desk.

All of your decisions and information are kept confidential. Your decisions can neither be observed by the players of other teams nor by us. Nor will you ever learn who your team mate is. Please respect other people's privacy and do not look at their smartphones.

On-Screen Instructions

The on-screen instructions can be found in Tables S1-S4. For some sample screens see the German version below.

Table S6. On-screen instructions for both players.

Number	Question Text	Type of Question	Options	Explanation
1	If you SHOOT, you have the chance to earn…	Single Choice	10€	Read the flyer carefully. If you want to start the game, please answer two comprehension questions on the game. Then you can start!
			40€	
			80€	
			160€	
2	If you SHOOT, you have the chance to earn…	Single Choice	10€	You provided the wrong answer. Please try again.
			40€	
			80€	
			160€	
3	If you PASS, …	Single Choice	the chance of scoring a goal decreases.	You answered the first question correctly. Please answer another question, then you can start.
			the chance of scoring a goal remains identical.	
			the chance of scoring a goal increases.	
4	If you PASS, …	Single Choice	the chance of scoring a goal decreases.	You provided the wrong answer. Please try again.
			the chance of scoring a goal remains identical.	
			the chance of scoring a goal increases.	
Continue with question 5 in Table S7 for right wing and in Table S8 for left wing.				

Notes: The numbers in brackets in column "Options" denote the number of the subsequent question if the participant decided in favor of that option.

Table S7. On-screen instructions for right wing player.

Num-ber	Question Text	Type of Question	Options	Explanation
5	You have the ball and you can SHOOT or PASS. If you SHOOT you score a goal with a probability of 5%. If you PASS the ball goes to left wing and he can decide. What do you do?	Single Choice	I SHOOT (6)<br>I PASS (7)	You answered the questions correctly. The game will start now.
6	If you had passed, do you think left wing would have SHOT at his 10% chance for a goal or would he have PASSED back to you?	Single Choice	Left wing would have SHOT (11)<br>Left wing would have PASSED (11)	You shot.
7	Do you think left wing will SHOOT at his 10% chance for a goal or will he PASS back to you?	Single Choice	Left wing will SHOOT (8)<br>Left wing will PASS (8)	You passed.
8	Assume that left wing PASSED at his chance for a goal of 10%. You can now SHOOT or PASS the ball. If you SHOOT you score a goal with a probability of 20%. If you PASS the ball goes to left wing and he can decide. What do you do?	Single Choice	I SHOOT (9)<br>I PASS (10)	Left wing is deciding whether to shoot or pass.
9	If you had passed, do you think, left wing would have SHOT at his 40% chance for a goal or would he have PASSED back to you?	Single Choice	Left wing would have SHOT (11)<br>Left wing would have PASSED (11)	You shot.
10	Do you think left wing will SHOOT at his 40% chance for a goal or will he PASS back to you?	Single Choice	Left wing will SHOOT (11)<br>Left wing will PASS (11)	You passed. Left wing is deciding whether to if he shoots or passes. If left wing passes, you shoot automatically.
Continue with question 11 in Table S9.				

Notes: The numbers in brackets in column "Options" denote the number of the subsequent question if the participant decided in favor of that option.

Table S8. On-screen instructions for left wing player.

Num-ber	Question Text	Type of Question	Options	Explanation
5	Right wing has the ball and he can SHOOT or PASS. If he SHOOTS he scores a goal with a probability of 5%. If he PASSES the ball goes to you and you can decide. What do you think, right wing will do?	Single Choice	Right wing will SHOOT (6)<br>Right wing will PASS (6)	You answered the questions correctly. The game will start now.
6	Assume that right wing PASSED at his chance for a goal of 5%. You can now SHOOT or PASS the ball. If you SHOOT you score a goal with a probability of 10%. If you PASS the ball goes to right wing and he can decide. What do you do?	Single Choice	I SHOOT (7)<br>I PASS (8)	
7	If you had passed, do you think right wing would have SHOT at his 20% chance for a goal or would he have PASSED back to you?	Single Choice	Right wing would have SHOT (11)<br>Right wing would have PASSED (11)	You shot.
8	Do you think, right wing will SHOOT at his 20% chance for a goal or will he PASS back to you?	Single Choice	Right wing will SHOOT (9)<br>Right wing will PASS (9)	You passed.
9	Assume that right wing PASSED at his chance for a goal of 20%. You can now SHOOT or PASS the ball. If you SHOOT you score a goal with a probability of 40%. If you PASS the ball goes back to right wing and he SHOOTS automatically. Thereby he scores a goal with a probability of 80%. What do you do?	Single Choice	I SHOOT (11)<br>I PASS (11)	Right wing is deciding whether to shoot or to pass.
Continue with question 11 in Table S9.				

Notes: The numbers in brackets in column "Options" denote the number of the subsequent question if the participant decided in favor of that option.

Table S9. Post-experimental questionnaire for both players

Num-ber	Question Text	Type of Question	Options
11	Thank you very much! All decisions were made. You will be told in the half-time break about how many goals were scored and whether your team was randomly selected to receive the payoffs. Please answer some questions for statistical purposes now.	Single Choice	I am male.
			I am female
12		Single Choice	I am less than 20 years old.
			I am between 20 and 30 years old.
			I am between 30 and 40 years old.
			I am more than 40 years old.
13	I am…	Single Choice	Pupil
			Studying
			Working
			Other
14	Did you discuss your decision with other smartphones users (participants)?	Single Choice	Yes
			No
15	Did you already participate at the game Shoot or Pass?	Single Choice	Yes
			No
16	What is the number on your table? (If you do not sit at a table you can leave the field empty.)	Input	Number of the table
17	I am…	Likert Scale	1 = sober
			5 = drunk
18	In general, I tend to…	Likert Scale	1 = avoid risks
			5 = take risks.
19	"An individual should subordinate himself/herself to the good of the community.“ With this statement…	Likert Scale	1 = I totally agree.
			5 = I totally disagree.
20	How are you feeling? At the moment I am…	Likert Scale	1 = relaxed / calm
			5 = aroused / tense
21	When Germany loses a World cup soccer game, I am…	Likert Scale	1 = not sad
			5 = very sad

Table S4. Final questionnaire for both players.

Sample Screens (original German version)

Figs S7 – S11 show some sample screens. For the full text of the on-screen instructions, see the English translation above or the original German version below. On the top of the screen, the role (left or right wing player) and the team (Germany, France, Argentina or none) were displayed. See Lambsdorff et al. (2017) for a publication on data where the team is identified. This publication on team reasoning refers only to data, where no information on a team is displayed.

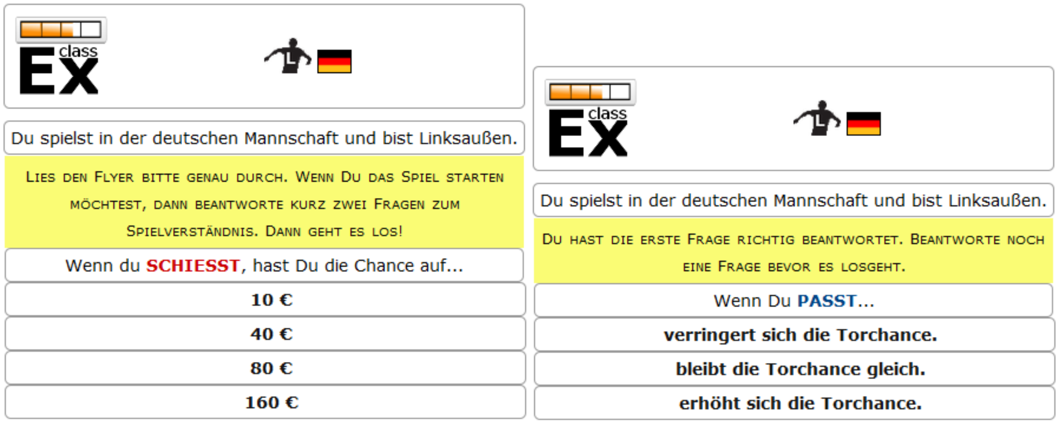

Fig S7. Comprehension questions

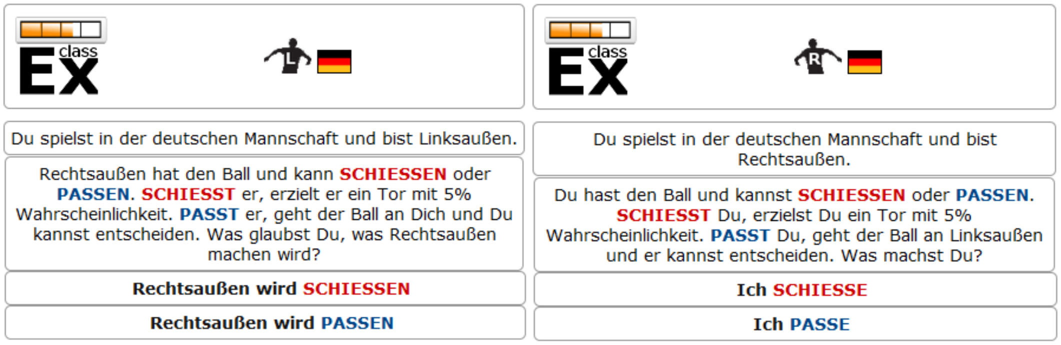

Fig S8. Decision screens

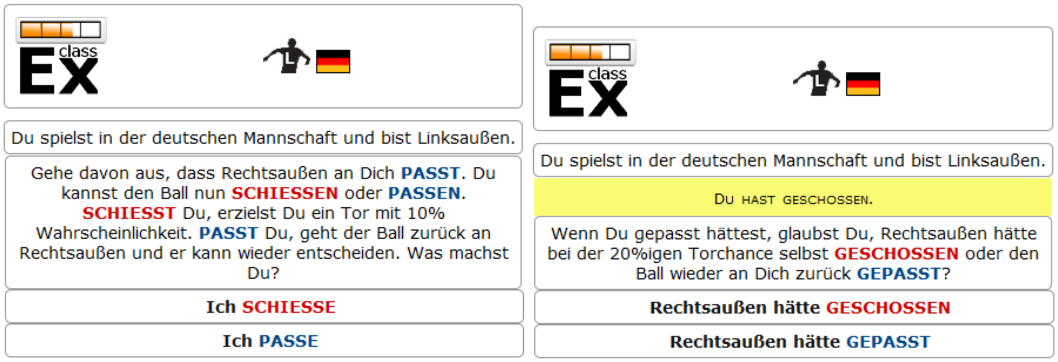

Fig S9. Expectation screens

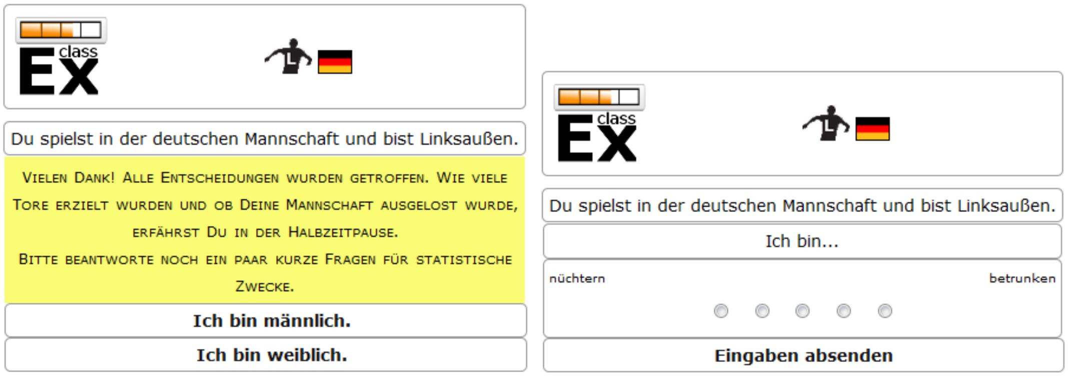

Fig S10. Post-experimental questionnaire I

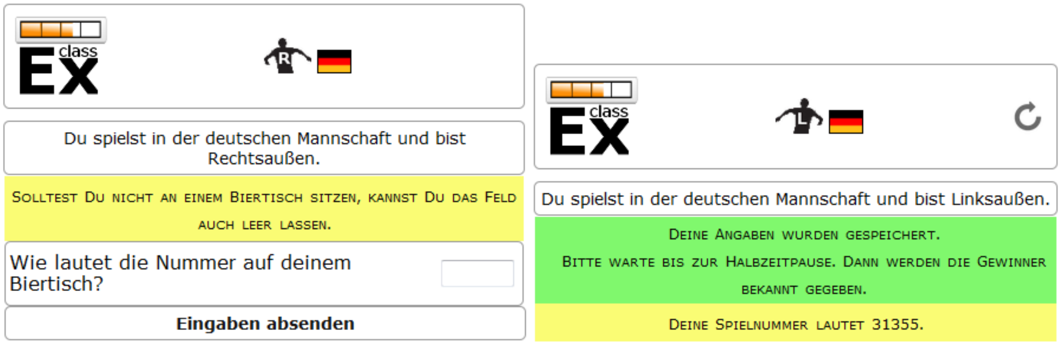

Fig S11. Post-experimental questionnaire II

Public Announcements (German version)

Servus zusammen und herzlich Willkommen zum Spiel SCHIESSEN oder PASSEN. Wenn ihr Fragen zum Spiel habt, könnt ihr euch jederzeit an unsere Helfer mit den gelben T-Shirts wenden.

Um an dem Spiel teilzunehmen, müsst Ihr den Flyer gut durchlesen und mit dem Smartphone auf die angegebene Internetseite gehen. Jeder kann mitspielen und als Torschütze 160€ oder als Mitspieler 40€ gewinnen.

[Wir haben das Spiel bereits zweimal an einem anderen Ort gespielt und insgesamt 2560€ ausgezahlt.] Heute können 3000€ insgesamt ausbezahlt werden. Das Spiel dauert etwa 5 Minuten. Es lohnt sich für Euch.

Sobald es losgeht, wird jeder von euch einer Mannschaft zugeteilt, die aus euch selbst und einem zufällig ausgewählten Mitspieler hier beim Public-Viewing hier im Hacklberg-Biergarten [in der Löwenbrauerei] besteht. Eure Position ist entweder Rechtsaußen oder Linksaußen. Ihr müsst mehrmals entscheiden zwischen SCHIESSEN **oder** PASSEN. Mit SCHIESSEN habt ihr die Chance auf die hohe Torprämie von 160€. Mit PASSEN erhöht sich die Torchance Eurer Mannschaft.

Das Spiel startet kurz vor dem Anpfiff des Viertelfinales Deutschland gegen Frankreich [Finales Deutschland gegen Argentinien]. Ihr habt Zeit bis zur 30 Minute des Viertelfinalspiels [Finalspiels]. Dann wird das Spiel von unserer Seite aus beendet.

Auf Eurem Smartphone wird Euch eine Spielernummer angezeigt. In der Halbzeitpause werde ich die Nummern der Gewinner bekanntgeben. Diese erhalten die Torprämie an unserem Infostand sofort in bar ausbezahlt. Besten Dank!

Written Instructions on Flyers (German version)

SCHIESSEN oder PASSEN?

Wir sind ein Team der Universität Passau und erheben Daten für Forschungszwecke. SCHIESSEN oder PASSEN startet kurz vor Anpfiff und geht bis zur 30 Minute des heutigen WM-Spiels. Du kannst mit Deinem Smartphone Entscheidungen treffen, ein Tor schießen und Geld verdienen. Jeder kann mitspielen und als Torschütze 160€ oder als Mitspieler 40€ gewinnen. Wenn Du mitmachen möchtest, lies die folgenden Anweisungen aufmerksam durch.

1. Vor Spielbeginn – Gut aufwärmen

Öffne kurz vor Anpfiff des WM-Spiels die Seite classEx.uni-passau.de/wm. Dort wirst Du einer Mannschaft und einer Position zugeteilt. Deine Mannschaft besteht aus Dir und einem zufällig ausgewählten Mitspieler. Deine Position ist entweder Rechtsaußen oder Linksaußen.

Fig S12. Written instructions page 1 (front of the leaflet)

2. Anpfiff – Der Angriff beginnt

Rechtsaußen (R) und Linksaußen (L) laufen auf das gegnerische Tor zu.

- Rechtsaußen hat den Ball und kann SCHIESSEN oder PASSEN. Falls er SCHIESST, erzielt er ein Tor mit 5% Wahrscheinlichkeit. Falls er PASST, geht der Ball an Linksaußen.
- Wenn Linksaußen den Ball bekommt, steht er ebenfalls vor der Entscheidung SCHIESSEN oder PASSEN. Mit SCHIESSEN erzielt er ein Tor mit 10% Wahrscheinlichkeit. Falls er PASST, geht der Ball an Rechtsaußen zurück.
- Wenn Rechtsaußen den Ball zurückbekommt, kann er erneut SCHIESSEN oder PASSEN. Falls er SCHIESST, erzielt er ein Tor mit 20% Wahrscheinlichkeit. Falls er PASST, geht der Ball erneut an Linksaußen.
- Wenn Linksaußen den Ball wieder bekommt, wählt er ein letztes Mal zwischen SCHIESSEN oder PASSEN. Falls er SCHIESST, erzielt er ein Tor mit 40% Wahrscheinlichkeit. Falls er PASST, geht der Ball an Rechtsaußen zurück, der optimal steht und ein Tor mit 80% Wahrscheinlichkeit erzielt.

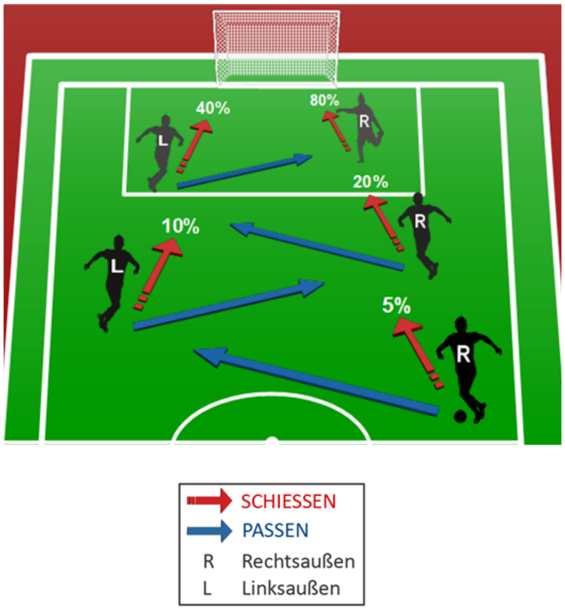

Fig S13. Written instructions pages 2 and 3 (inside of the leaflet)

Im Anschluss sind noch ein paar Fragen für statistische Zwecke zu beantworten.

3. Abpfiff – Hast Du gewonnen?

Per Zufallsverfahren werden nach Spielende unter allen Teilnehmern 10 Mannschaften ausgewählt. Diese erhalten die Torprämien ausbezahlt, sofern sie ein Tor geschossen haben. Der Torschütze erhält 160€, der Mitspieler 40€.

Jeder Spieler erhält eine Spielernummer, die am Ende des Spiels auf dem Smartphone angezeigt wird. Verliere diese Nummer nicht. In der Halbzeitpause lesen wir die Spielernummern vor, die gewonnen haben. Wenn Du gewonnen hast, wird Dir gegen Vorlage Deiner Spielernummer die Torprämie an unserem Infostand sofort in bar ausbezahlt.

Alle Deine Entscheidungen und Informationen bleiben anonym. Deine Entscheidungen können weder von Spielern anderer Mannschaften noch von uns beobachtet werden. Auch erfährst Du nicht, wer Dein Mitspieler ist. Bitte respektiere die Privatsphäre anderer und schaue nicht auf ihre Smartphones.

Fig S14. Written instructions page 4 (back of the leaflet)

Table S10. On-Screen Instructions for both players

Num-mer	Fragentext	Fragentyp	Optionen	Erläuterung
1	Wenn du SCHIESST, hast Du die Chance auf...	Single Choice	• 10€<br>• 40€<br>• 80€<br>• 160€	Lies den Flyer bitte genau durch. Wenn Du das Spiel starten möchtest, dann beantworte kurz zwei Fragen zum Spielverständnis. Dann geht es los!
2	Wenn du SCHIESST, hast Du die Chance auf...	Single Choice	• 10€<br>• 40€<br>• 80€<br>• 160€	Du hast die Frage falsch beantwortet. Probiere es nochmal.
3	Wenn Du PASST...	Single Choice	• verringert sich die Torchance.<br>• bleibt die Torchance gleich.<br>• erhöht sich die Torchance.	Du hast die erste Frage richtig beantwortet. Beantworte noch eine Frage, bevor es losgeht.
4	Wenn Du PASST...	Single Choice	• verringert sich die Torchance.<br>• bleibt die Torchance gleich.<br>• erhöht sich die Torchance.	Du hast die Frage falsch beantwortet. Probiere es nochmal.
Weiter mit Frage 5 in Table S11 für Rechtsaußen und Table S12 für Linksaußen.				

Table S11. On-Screen Instructions for right wing player.

Nummer	Fragentext	Fragentyp	Optionen	Erläuterung
5	Du hast den Ball und kannst SCHIESSEN oder PASSEN. SCHIESST Du, erzielst Du ein Tor mit 5% Wahrscheinlichkeit. PASST Du, geht der Ball an Linksaußen und er kann entscheiden. Was machst Du?	Single Choice	Ich SCHIESSE (6)<br>Ich PASSE (7)	Du hast die Fragen richtig beantwortet. Das Spiel geht jetzt los.
6	Wenn Du gepasst hättest, glaubst Du, Linksaußen hätte bei seiner 10% Torchance GESCHOSSEN oder GEPASST?	Single Choice	Linksaußen hätte GESCHOSSEN (11)<br>Linksaußen hätte GEPASST (11)	Du hast geschossen.
7	Glaubst Du, Linksaußen wird bei seiner 10% Torchance SCHIESSEN oder den Ball an Dich zurück PASSEN?	Single Choice	Linksaußen wird SCHIESSEN (8)<br>Linksaußen wird PASSEN (8)	Du hast gepasst.
8	Gehe davon aus, dass Linksaußen bei seiner 10% Torchance GEPASST hat. Du kannst den Ball nun SCHIESSEN oder PASSEN. SCHIESST Du, erzielst Du ein Tor mit 20% Wahrscheinlichkeit. PASST Du, geht der Ball zurück an Linksaußen und er kann entscheiden. Was machst Du?	Single Choice	Ich SCHIESSE (9)<br>Ich PASSE (10)	Linksaußen entscheidet, ob er schießt oder passt.
9	Wenn du gepasst hättest, glaubst Du, Linksaußen hätte bei seiner 40% Torchance GESCHOSSEN oder den Ball wieder an Dich zurück GEPASST?	Single Choice	Linksaußen hätte GESCHOSSEN (11)<br>Linksaußen hätte GEPASST (11)	Du hast geschossen.
10	Glaubst Du, Linksaußen wird bei seiner 40% Torchance SCHIESSEN oder den Ball wieder an Dich zurück PASSEN?	Single Choice	Linksaußen wird SCHIESSEN (11)<br>Linksaußen wird PASSEN (11)	Du hast gepasst. Linksaußen entscheidet, ob er schießt oder passt. Falls Linksaußen passt, schießt du automatisch.
Weiter mit Frage 11 in Table S13.				

Anmerkung: Die Zahl in Klammern in der Spalte "Optionen" gibt die Nummer der folgenden Frage an, falls sich ein Teilnehmer für diese Option entschieden hat).

Table S12. On-screen instructions for left wing player.

Nummer	Fragentext	Fragentyp	Optionen	Erläuterung
5	Rechtsaußen hat den Ball und kann SCHIESSEN oder PASSEN. SCHIESST er, erzielt er ein Tor mit 5% Wahrscheinlichkeit. PASST er, geht der Ball an Dich und du kannst entscheiden. Was glaubst Du, was Rechtsaußen machen wird?	Single Choice	Rechtsaußen wird SCHIESSEN (6)<br>Rechtsaußen wird PASSEN (6)	Du hast die Fragen richtig beantwortet. Das Spiel geht jetzt los.
6	Gehe davon aus, dass Rechtsaußen bei seiner 5% Torchance GEPASST hat. Du kannst den Ball nun SCHIESSEN oder PASSEN. SCHIESST Du, erzielst Du ein Tor mit 10% Wahrscheinlichkeit. PASST Du, geht der Ball zurück an Rechtsaußen und er kann entscheiden. Was machst Du?	Single Choice	Ich SCHIESSE (7)<br>Ich PASSE (8)	Du hast geschossen.
7	Wenn Du gepasst hättest, glaubst Du, Rechtsaußen hätte bei seiner 20% Torchance GESCHOSSEN oder den Ball wieder an Dich zurück GEPASST?	Single Choice	Rechtsaußen hätte GESCHOSSEN (11)<br>Rechtsaußen hätte GEPASST (11)	Du hast gepasst.
8	Glaubst Du, Rechtsaußen wird bei seiner 20% Torchance SCHIESSEN oder an Dich zurück PASSEN?	Single Choice	Ich SCHIESSE (9)<br>Ich PASSE (9)	Linksaußen entscheidet, ob er schießt oder passt.
9	Gehe davon aus, dass Rechtsaußen bei seiner 20% Torchance GEPASST hat. Du kannst den Ball nun SCHIESSEN oder PASSEN. SCHIESST Du, erzielst Du ein Tor mit 40% Wahrscheinlichkeit. PASST Du, geht der Ball wieder zurück an Rechtsaußen und er SCHIESST automatisch. Damit erzielt er ein Tor mit 80% Wahrscheinlichkeit. Was machst Du?	Single Choice	Ich SCHIESSE (11)<br>Ich PASSE (11)	Du hast geschossen.
Weiter mit Frage 11 in Table S13.				

Anmerkung: Die Zahl in Klammern in der Spalte “Optionen” gibt die Nummer der folgenden Frage an, falls sich ein Teilnehmer für diese Option entschieden hat).

Table S13. Post-experimental questionnaire for both players.

11		Vielen Dank! Alle Entscheidungen wurden getroffen. Wie viele Tore erzielt wurden und ob Deine Mannschaft ausgelost wurde, erfährst Du in der Halbzeitpause. Bitte beantworte noch ein paar kurze Fragen für statistische Zwecke.	Single Choice	Ich bin männlich.
				Ich bin weiblich.
12			Single Choice	Ich bin unter 20 Jahre a
				Ich bin 20-30 Jahre alt.
				Ich bin 30-40 Jahre alt.
				Ich bin über 40 Jahre al
13		Ich bin…	Single Choice	Schüler
				Student
				Berufstätig
				Anderes
14		Hast Du Deine Entscheidungen im Spiel mit anderen Smartphone-Nutzern (Teilnehmern am Biergartenspiel) abgestimmt?	Single Choice	Ja
				Nein
15		Hast Du schon einmal am Spiel SCHIESSEN oder PASSEN teilgenommen?	Single Choice	Ja
				Nein
16		Was ist Deine Tischnummer (Falls Du nicht an einem Tisch sitzt, kannst Du das Feld leer lassen).	Input	Tischnummer
17		Ich bin…	Likert Skala	1 = nüchtern
				5 = betrunken
18		Im Allgemeinen neige ich dazu, Risiken...	Likert Skala	1 = zu vermeiden
				5 = auf mich zu nehmen
19		"Der Einzelne sollte sich dem Wohl der Gemeinschaft unterordnen." Dieser Aussage stimme ich...	Likert Skala	1 = voll und ganz zu
				5 = überhaupt nicht zu
20		Wie fühlst du dich? Ich bin gerade...	Likert Skala	1 = ruhig / entspannt
				5 = aufgeregt / gespann
21		Wenn Deutschland ein WM-Spiel verliert, bin ich…	Likert Skala	1 = nicht traurig
				5 = sehr traurig
